# Leveraging community mortality indicators to infer COVID-19 mortality and transmission dynamics in Damascus, Syria

**DOI:** 10.1038/s41467-021-22474-9

**Published:** 2021-04-22

**Authors:** Oliver J. Watson, Mervat Alhaffar, Zaki Mehchy, Charles Whittaker, Zack Akil, Nicholas F. Brazeau, Gina Cuomo-Dannenburg, Arran Hamlet, Hayley A. Thompson, Marc Baguelin, Richard G. FitzJohn, Edward Knock, John A. Lees, Lilith K. Whittles, Thomas Mellan, Peter Winskill, Imperial College COVID-19 Response Team, Samir Bhatt, Bimandra A. Djaafara, Christl A. Donnelly, Seth Flaxman, Katy A. M. Gaythorpe, Natsuko Imai, Elita Jauneikaite, Daniel J. Laydon, Swapnil Mishra, H. Juliette T. Unwin, Robert Verity, Natasha Howard, Hannah Clapham, Francesco Checchi, Neil Ferguson, Azra Ghani, Emma Beals, Patrick Walker

**Affiliations:** 1 MRC Centre for Global Infectious Disease Analysis, Jameel Institute for Disease and Emergency Analytics, Imperial College London, London, UK; 2 Department of Infectious Disease Epidemiology, Faculty of Epidemiology and Population Health, London School of Hygiene and Tropical Medicine, London, UK; 3 Syria Team, Conflict Research Programme, London Schools of Economics, London, UK; 4 Google Cloud Developer Advocacy, Google, London, UK; 5 Saw Swee Hock School of Public Health, National University of Singapore and National University Health System, Singapore, Singapore; 6 European Institute of Peace, Brussels, Belgium; 7 Middle East Institute, Washington, DC USA

**Keywords:** Computational models, SARS-CoV-2, Epidemiology, Developing world

## Abstract

The COVID-19 pandemic has resulted in substantial mortality worldwide. However, to date, countries in the Middle East and Africa have reported considerably lower mortality rates than in Europe and the Americas. Motivated by reports of an overwhelmed health system, we estimate the likely under-ascertainment of COVID-19 mortality in Damascus, Syria. Using all-cause mortality data, we fit a mathematical model of COVID-19 transmission to reported mortality, estimating that 1.25% of COVID-19 deaths (sensitivity range 1.00% – 3.00%) have been reported as of 2 September 2020. By 2 September, we estimate that 4,380 (95% CI: 3,250 – 5,550) COVID-19 deaths in Damascus may have been missed, with 39.0% (95% CI: 32.5% – 45.0%) of the population in Damascus estimated to have been infected. Accounting for under-ascertainment corroborates reports of exceeded hospital bed capacity and is validated by community-uploaded obituary notifications, which confirm extensive unreported mortality in Damascus.

## Abstract (web summary)

Reported COVID-19 mortality rates have been relatively low in Syria, but there has been concern about overwhelmed health systems. Here, the authors use community mortality indicators and estimate that <3% of COVID-19 deaths in Damascus were reported as of 2 September 2020.

## Introduction

The COVID-19 pandemic is a major global threat, with 25,327,098 cases and 848,255 deaths confirmed as of 1 September 2020^1^. The majority of countries have responded to the threat by implementing various non-pharmaceutical interventions, with many opting for society-wide suppression measures in an effort to prevent unacceptable loss of life^2^. However, a number of conflict-affected countries in Africa and West Asia, e.g. Sudan, Syria, and Yemen, have reported no sharp increases in mortality of the scale predicted by multiple modelling studies, even where interventions were limited^3,4^. These observations are hard to reconcile with contemporaneous reports of hospitals becoming overwhelmed in these settings^5,6^. Additionally, many settings with very low observed mortality are now reporting that cases and hospital bed demand are falling despite no notable change in implemented interventions or behaviour change, which may suggest that the epidemic has passed its peak^7^. Consequently, alternative explanations have been sought to understand the heterogeneity in the magnitude of COVID-19 epidemics, including the effects of climate^8^, population density^9^, inter-pathogen effects^10^, younger populations^11^ and BCG vaccination^12^ on COVID-19 transmission or clinical severity.

One alternative (or concurrent) explanation, which has garnered less attention within the scientific literature despite numerous reports in the media^13,14^, is that COVID-19 related deaths have been substantially under-ascertained in some countries. Under-ascertainment of COVID-19 symptomatic cases has been recognised as an issue in many high and low-income settings, due to factors such as limited testing capacity and the non-specific symptoms of mild disease^15^. Because clinically severe cases are more likely to be recognised and tested^16^, mortality data have been viewed as a more reliable datastream for cross-country comparisons and for tracking epidemics^17^. However, under-ascertainment of COVID-19 deaths is known to occur, with investigations comparing excess mortality and reported lab-confirmed COVID-19 deaths revealing substantial discrepancies, particularly at the beginning of epidemics and at the peak of transmission^14,18^. Consequently, all-cause, excess population mortality has been considered the most reliable data for comparing the magnitude and trajectory of COVID-19 epidemics across countries^19^. Unfortunately, real-time estimates of all-cause mortality are unavailable for many conflict-affected countries where comprehensive vital registration systems may be lacking or poorly-functioning and data sharing may be restricted^20^.

Motivated by reports of an overwhelmed health system^21^ and widespread under-ascertainment of deaths^22^ we sought to understand the evolving COVID-19 epidemic in Syria, a former middle-income country, now classified as low-income by the World Bank for 2020–2021 after the destructive ongoing conflict. Before 2011, health indicators in Syria had improved considerably and the country had begun its epidemiological transition with 77% of mortality due to non-communicable diseases^23^. However, pre-conflict health system challenges surrounding among other issues, data validity and transparency, inadequate healthcare provider coordination and staffing levels as well as uncontrolled private-sector expansion, all contributed to uneven regional health services distribution^23^. The March 2011 uprising, violent government response, and escalated armed conflict since mid-2012 is well documented^24^. Waves of population displacement has meant an estimated 11.7 million people in Syria needed humanitarian health assistance in 2019^24,25^. The protracted conflict, entailing dynamic shifts in conflict lines and political boundaries, has fragmented the country’s governance among opposing military forces including the Syrian government, opposition groups, Syrian Democratic forces, and Turkish forces^24^. This has fragmented the country’s governance and led to shortages of supplies and equipment, systematic targeting of healthcare facilities and staff and the forced migration of skilled healthcare professionals. These issues have contributed to an increasingly fragmented and politicised health system^26,27^ that reflects a compromised response to health needs but in which essential accountability, transparency, and information sharing across areas-of-control is only possible with external support^26^. This study contributes analysis of a novel data source to improve transparency within Syria, support greater accountability and demonstrate an approach that could be used for similar research in other conflict-affected and resource-constrained settings.

## Results

### Estimating the under-ascertainment of deaths in the first wave in Damascus

In this study, we focus on reports from the capital city, Damascus, where 832 deaths were recorded by the mortuary office between 25 July and 1 August, an average of 104 per day (Supplementary Table 3)^28^. All-cause mortality data is not routinely published and the 8 days of data were only published by the mortuary office in response to public pressure for transparency. The reported deaths are significantly in excess of estimates of pre-pandemic expected daily mortality, namely 32 deaths per day given annual mortality for Damascus reported by the Syrian Central Bureau of Statistics^29^. One week earlier, the director of the Damascus office for burial of the dead suggested that the pre-pandemic daily mortality in Damascus during this period of the year was ‘~40 deaths, a normal figure in the summer as deaths increase due to high heat’^30^. This estimate is in line with reports from 2016 by the same director that daily deaths are in the range of 15–50 deaths per day^31^. Applying these broadly consistent pre-pandemic baselines, we compute approximate excess mortality by subtracting an assumed baseline mortality of 32 deaths per day, which we use to infer the level of under-ascertainment of COVID-19 deaths in Damascus prior to 2 September. This estimate of excess mortality is only an approximate one and may underestimate true excess mortality if the deaths reported between 25 July and 1 August are themselves an under-ascertainment. In addition, we consider a higher baseline mortality of 64 deaths per day to explore increased indirect effects of COVID-19 on mortality.

We fit a previously published^3^ age-structured COVID-19 transmission model to the official reported daily COVID-19 deaths in Damascus governorate (Supplementary Table 4). The model is a population-based age-structured Susceptible-Exposed-Infected-Recovered model, which explicitly represents disease severity and passage through different healthcare levels (Supplementary Methods). To infer changes in transmission, we estimate the impact of implemented non-pharmaceutical interventions in Damascus (Supplementary Table 5), which started on 13 March with most interventions being relaxed on 26 May. To estimate the under-ascertainment of COVID-19 deaths, we assume that a constant proportion of the model-predicted COVID-19 deaths on a given day are ascertained and reported. Using this assumption, the trajectory of official reported deaths should mirror the shape of the epidemic after accounting for under-ascertainment. We scan across a range of under-ascertainment levels to relate the deaths predicted by the model to official reported deaths. We identify the most likely level of mortality under-ascertainment by comparing the total predicted COVID-19 deaths between 25 July and 1 August from the transmission model against the excess mortality estimated from government-reported all-cause mortality data in Damascus for the same period. Given the extensive changes to Syrian demography and health services resulting from war, and ongoing uncertainty regarding key COVID-19 parameters, we conducted an extensive sensitivity analysis, exploring the impact that the assumed demography, population size in Damascus, healthcare capacity, infection fatality ratios (IFRs) and baseline daily mortality have on the estimated level of under-ascertainment (Supplementary Table 6 for all model inputs explored).

**Fig. 1:**
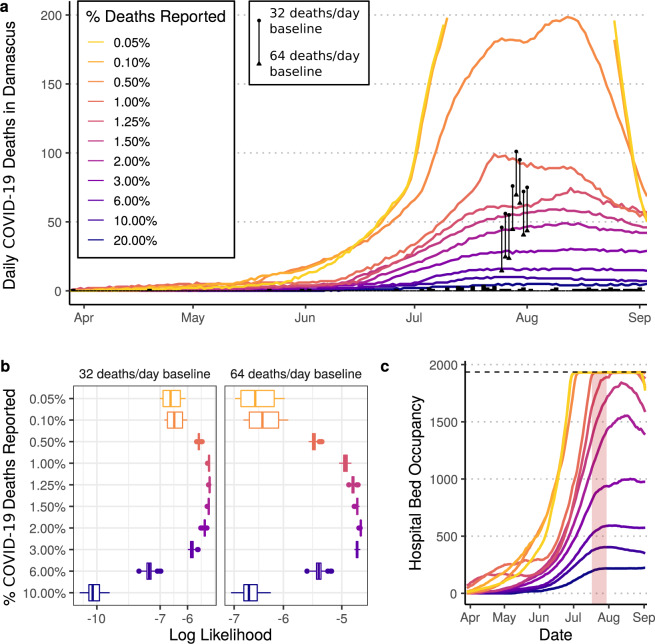
Estimates of under-ascertainment of deaths in Damascus. In (**a**) the daily model-predicted COVID-19 deaths in Damascus are shown using the default model parameters and a range of values for the under-ascertainment of COVID-19 deaths. These are compared to the excess mortality reported by the government during 25 July–1 August, which is shown with point ranges, with estimates assuming a baseline of 32 and 64 deaths per day shown with a circle and triangle respectively. Trajectories with 0.05 and 0.1% of deaths reported are not shown above 200 daily deaths to ease viewing of other curves. In (**b**) the log likelihood for each level of under-ascertainment is shown for different assumed baseline mortality. Model log likelihoods presented reflect the mean model log likelihood across the full sensitivity analysis conducted (*n* = 14,000), suggesting that under-ascertainment is likely between 1 and 3% when viewed across both baseline mortality estimates. The box represents the median and interquartile range (IQR) and whiskers represent the IQR ± 1.5*IQR with points depicting values outside this range. In (**c**) the model-predicted hospital occupancy for the simulations in (**a**) are shown. The hospital capacity for Damascus is shown with a dashed horizontal line, with the 2-week period in which hospitals were reported to be first at capacity shown shaded in red. In both (**a**, **c**) the median of 100 draws from the posterior parameter space are presented for visualisation purposes.

As of 2 September 2020, 60 COVID-19 related deaths were reported in Damascus governorate (Supplementary Fig. 3). We estimate that this comprises 1.25% of total COVID-19 deaths, with an estimated 4440 COVID-19 deaths (95% CI: 3310–5600) by 2 September 2020 (Fig. 1). This was determined by comparing the model-predicted COVID-19 deaths between 25 July and 1 August to the excess mortality under the assumption that all excess deaths are due to COVID-19 (Fig. 1a). If we double our assumed baseline death rate from 32 to 64 deaths per day, we still estimate substantial under-ascertainment with an estimated 2% of deaths due to COVID-19 being reported as such (Fig. 1b). To explore the reliability of these model fits, we also looked at reports of hospitals reaching capacity in Damascus, resulting in patients being treated at home. These reports vary, but there is consensus that individuals were reluctant to go to hospitals between 17 July^32^ and 30 July^33^ due to hospitals reaching capacity and having to turn away patients. With our default parameters, we predict that hospital capacity would have been reached during this period with an assumed under-ascertainment of deaths between 1 and 1.25% (Fig. 1c). This lends further support to our inferred most likely under-ascertainment of 1.25%. This finding is dependent on the assumed number of hospital beds available, which is unknown due to uncertainty in the number of functional beds across both public and private hospitals. However, in our sensitivity analysis the best model fit to mortality data was obtained assuming ~2000 hospital beds were available, consistent with the value of 1935 beds used to generate our central estimates (Supplementary Fig. 4). Sensitivity analysis showed our estimates to be robust to varying assumptions about the population size and demography of the Damascus population. However, we obtained a better model fit when we assumed a higher IFR than given by the Verity et al. estimates^34^. Higher IFRs are plausible if access to oxygen support was limited at the peak of the epidemic^3^. This is unknown, however, anonymous testimonials from Damascus report individuals trying to buy their own oxygen during the peak in transmission suggesting^35^. Looking across the full range of assumptions examined in the sensitivity analysis, we estimate that between 1 and 3% of deaths due to COVID-19 were ascertained and reported.

The excess mortality recorded between 25 July and 1 August provided a small window to analyse the COVID-19 epidemic in Damascus. Given the limited amount of data, it is hard to be confident in the predicted epidemic dynamics. For example, in Fig. 1c, the model predicts that the demand for hospital beds started to decrease by the end of August. However, there are multiple possible epidemic trajectories that could yield the number of excess deaths reported. In Supplementary Fig. 5, we explore three alternative scenarios and their compatibility with the observed data. We identified one of these (Supplementary Fig. 5D)—a scenario assuming that only COVID-19 deaths occurring in hospitals are reported—as showing a comparably good fit to all available data (i.e. reported daily deaths, excess deaths and the timing of the health system reaching capacity). This alternative scenario would imply 0.28% (95% CI: 0.24–0.39%) of total COVID-19 deaths are reported (Supplementary Fig. 6) and predicts a significantly larger number of excess deaths compared to the original analysis, with 21,260 deaths (95% CI: 15,360–24,860) estimated by 2 September 2020 (Supplementary Fig. 7). Given the approximate fivefold increase in estimated deaths compared to the earlier estimate of 4400 deaths, and without the ability to verify whether reported deaths encompass both hospital and community deaths, we sought to gain an alternative source of data to confirm mortality trends in Damascus.

### Estimating excess mortality in Damascus from obituary notifications

**Fig. 2:**
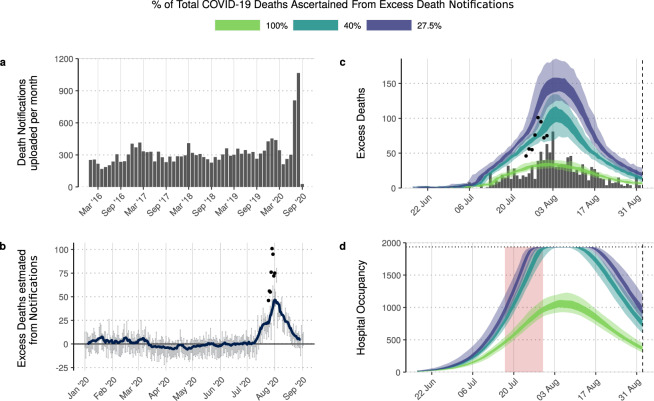
Community-uploaded death notifications in Damascus and the inferred epidemic trajectory. In (**a**) we show the number of obituary notification photos uploaded monthly to the community maintained ‘Damascus Mortality’ Facebook page revealing a substantial increase in July and August 2020. This is in contrast to the consistent monthly number of notifications uploaded between 2016 and 2020. In (**b**), the daily inferred excess death notifications are shown, with error bars showing the 95% CI (*n* = 100) and the blue line indicating the weekly average excess deaths. In (**c**) model fits to the notification excess death data (vertical bars) are shown under different assumptions of the proportion of total COVID-19 deaths that are ascertained by the notification excess deaths. In both (**b**, **c**) the points show the government-reported excess deaths between 25 July and 1 August. The predicted hospital occupancy is shown in (**d**), with the hospital capacity for Damascus shown with a dashed horizontal line, with the 2-week period in which hospitals were reported to be first at capacity shown shaded in red. In (**c**, **d**) the 50% and 95% CI are shown with dark and light shaded regions respectively.

Traditionally, when individuals die in Damascus, a paper notification of death is printed and affixed to household walls in nearby neighbourhoods of the deceased. These notifications include information, such as the date of death, details of the deceased’s relatives and the consolation events being held. However, due to internal displacements and a high number of Syrians leaving the country due to recent conflict, a community maintained Facebook page (‘Damascus Mortality’^36^) was established to inform people about deaths in Damascus. On this page, images of the notifications produced by printing shops in Damascus are routinely uploaded with the majority of images uploaded within 24 h of the noted date of death. From analysing the noted date of death, all images are uploaded within 5 days after death, with a mean delay of 0.49 days. Despite the informality of this process, the observed mortality trends in this alternative data source reveal a consistent baseline of ~300 notifications per month in 2017–2019 (Fig. 2a), which represents 27.5% of annual reported mortality. By contrast, uploaded notifications rose to 809 in July and 1066 in August 2020.

To obtain an additional view of the epidemic in Damascus, we separately fitted our transmission model to the notification data. We calculated the excess notification deaths for 2020 by subtracting the mean number of notifications uploaded daily in 2017–2019 for each month from those reported in 2020. The inferred estimates suggest that excess deaths increased from the beginning of July, with a clear peak at the end of July (Fig. 2b). We therefore fitted the model to excess death notifications from July onwards, using three approaches to reflect the uncertainty in the fraction of total COVID-19 deaths captured by the excess death notifications. For our baseline model for fitting to death notifications, we assumed that excess deaths recorded within online death notifications were a constant proportion of underlying excess deaths recorded by the Damascus Mortuary Office between 25 July and 1 August (best fitted by an ascertainment rate within the online death notifications of 40%, i.e. for every 2 death notifications there were five all-cause deaths).

Using the baseline model for death notifications, we estimate 2920 COVID-19 deaths (95% CI: 2480–3460) have occurred by 2 September 2020, which equates to 2% of COVID-19 deaths in Damascus being reported. This estimate falls within the sensitivity range for the estimate of under-ascertainment of 1–3% estimated earlier in Fig. 1. We also explored a maximum upper and lower ascertainment fraction, either assuming that 100% of COVID-19 deaths are captured by the excess death notifications or that only 27.5% of COVID-19 deaths are captured by the notifications, which reflects the proportion of total all-cause mortality in Damascus that was captured by the notifications in 2017–2019^29^. This produced upper and lower estimates of ascertainment of deaths in official COVID-19 reports of 1.5% and 5% respectively. The upper estimate of 5%, however, is unlikely to be reflective of the situation in Damascus, with estimated hospital bed demand never exceeding capacity (Fig. 2d). The lower estimate, by contrast, is close to our best-fitting model in Fig. 1, in which 1.25% of deaths were ascertained, resulting in health systems reaching capacity as reported at the end of July. The excess death notification data thus provides supporting evidence that both substantial under-reporting of COVID-19 deaths has occurred and confirms the recent declines in transmission predicted when fitting to reported daily deaths has indeed occurred in Damascus.

### Estimates of COVID-19 epidemic spread during first wave in Damascus

**Fig. 3:**
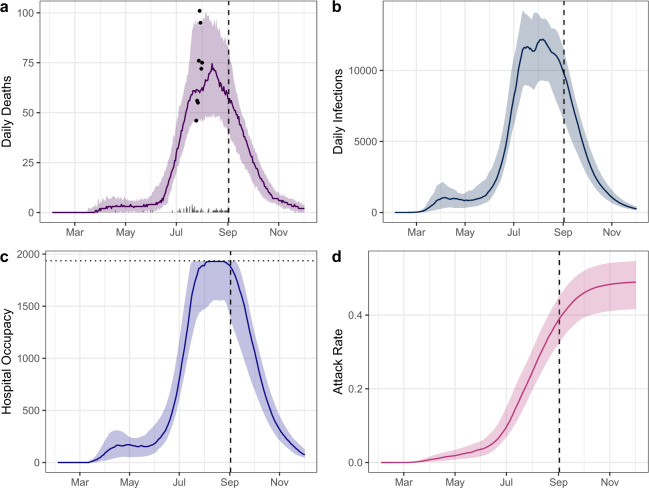
Model-predicted deaths, infections, hospital occupancy and attack rates of COVID-19 for Damascus. The best-fitting model to excess deaths (assumed baseline mortality as 32 deaths per day) is shown, which estimates that 1.25% of deaths are reported. In (**a**, **b**) the reported daily deaths and infections due to COVID-19 respectively are shown, with the estimated excess deaths for a baseline mortality of 32 deaths per day shown in (**a**) as points. A short delay is observed between infections in (**a**) and deaths in (**b**) reflecting the decrease in the delay from infection to death when there are insufficient hospital and ICU beds. In (**c**) hospital occupancy over time is shown, with the dotted horizontal line showing the hospital capacity available. In (**d**) the attack rate in Damascus is shown. In all plots, the median trajectory and 95% confidence interval (shaded region) is shown. A vertical dashed line is shown for 2 September 2020 when the analysis was conducted. The 3-month projection assuming the current level of transmission is shown in each plot.

The scale of inferred under-ascertainment indicates a starkly different epidemic from that suggested by reported deaths. At the time of analysis (2 September) this had profound implications for the future trajectory of the epidemic. To reconstruct the course of the epidemic to date and forecast its future trajectory, we selected the best-fitting model from our default parameter set, which estimated that 1.25% of COVID-19 deaths are ascertained, and projected it forward for 90 days, assuming that interventions and population contact patterns remain constant after 2 September 2020. Of the 4440 deaths we estimate to have occurred in Damascus up to 2 September 2020 (Fig. 3a), 3820 COVID-19 deaths (95% CI: 2800–4800) are predicted to have occurred between 1 July and 2 September 2020, comparable to the estimated 4200 COVID-19 deaths (95% CI: 3710–5000) made when fitting to the Facebook obituary deaths and assuming that excess death notifications represent 27.5% of the total COVID-19 deaths. We estimate that there were 67,290 (95% CI: 44,610–77,910) currently infected individuals in Damascus on 2 September 2020. 9760 (95% CI: 6470–11,360) individuals were estimated to be infected on 2 September 2020, including both asymptomatic and symptomatic infections. Consequently, we predict that a cumulative total of 39.0% (95% CI: 32.5–45.0%) of the population in Damascus (2,394,000) had been infected by 2 September 2020 (Fig. 3d).

Assuming that *R*_t_ remained constant after 2 September, we predict that the epidemic has passed its transmission peak, in agreement with the peak observed in Facebook death notification data. We estimate that 240 (95% CI: 170–280) new COVID-19 cases requiring hospital-level health interventions occurred on 2 September 2020, with the peak in hospitalisation requirement predicted to have occurred 1 month earlier during the first week of August 2020. However, despite the decrease in admissions, we predict that there were insufficient hospital beds for the majority of August 2020, with beds becoming available again from 28th August 2020 (Fig. 3c). We predict that the indicators of declines in transmission observed in Damascus will continue, with 49.1% (95% CI: 41.7–54.7%) of the population being infected by the end of 2020. We estimate that the effective reproduction number, *R*_eff_, for Damascus was 0.87 (95% CI: 0.82–0.92) on 2 September 2020 (green curves in Supplementary Fig. 8a). In the absence of immunity, we estimate that *R*_t_ (the reproduction number unadjusted for population immunity), would have been 1.48 (95% CI: 1.34–1.65) on 2 September 2020 (blue curves in Supplementary Fig. 8a). The decline in transmission in Damascus observed by 2 September 2020 thus appears largely due to increasing recovery-related immunity in the population and the resultant reduction of susceptible individuals, with 40% of the population estimated to have been infected by 2 September. The value of *R*_t_ of 1.48 on 2 September 2020 is significantly lower than the estimate of R_0_, which was estimated to be 2.77 (95% CI: 2.45–2.94). Given the absence of major public health interventions in place on 2 September 2020, we attribute the majority of reductions in *R*_t_ (i.e. decreases in transmission not due to immunity) to be due to individual behavioural changes.

### Model predictive performance and characterisation of Damascus second wave

A notable challenge exists in conducting research in settings with weakened health system governance. In conducting this analysis, a number of authors of this study requested to be acknowledged anonymously for security reasons (see Acknowledgements section for their contributions as authors). The methods taken to contact and collaborate safely on this research with these authors introduced understandable delays, highlighting the need for research findings to be communicated quickly through scientific reports and preprint servers^37^. The results presented so far reflect analysis that was conducted during September 2020. However, during revision of this manuscript, a notable increase in COVID-19 cases and deaths was recorded in Syria starting at the end of November. We sought to incorporate these data on revision.

**Fig. 4:**
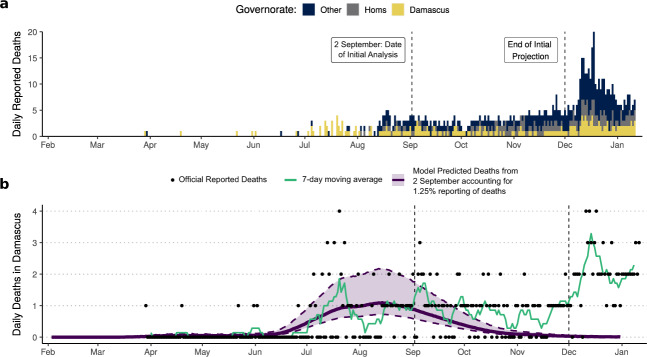
Predictive performance of second wave dynamics in Damascus. In (**a**) the official reported COVID-19 deaths in Syria are shown, with deaths in Damascus (gold) and Homs (grey) governorates highlighted indicating the shifting burden COVID-19 has placed on regions outside of Damascus. In (**b**) the performance of model projections made on 2 September (purple, median and 95% CI in shaded region) are shown compared against officially reported COVID-19 deaths (black points) and the weekly rolling mean (green). Model predictions correctly suggested a decrease in COVID-19 deaths in Damascus after the peak during the Summer, however, they failed to predict an increase in reported COVID-19 deaths towards the end of November.

Between 2 September and the end of November, reported COVID-19 deaths in Damascus decreased, with the weekly average remaining below the peak seen during mid July (Fig. 4a). The decrease in reported deaths aligned with the model-predicted deaths that would have been reported after accounting for the earlier estimated fraction of COVID-19 that are reported (1–3%) (Fig. 4b). However, during this period, the epidemic was observed to have spread to other governorates in Syria with the majority of deaths occurring outside of Damascus, in particular in the Homs governorate region (Fig. 4a). After December, a substantial increase in COVID-19 mortality was observed in all governorates, with numerous media reports of stretched healthcare systems in other governorates^38^. In order to understand the drivers of the second wave observed in Damascus, we sought to leverage the death notification data to estimate the proportion of COVID-19 deaths during the second wave in Damascus and determine if the dynamics were due to increased transmission or increased reporting of COVID-19 mortality.

**Fig. 5:**
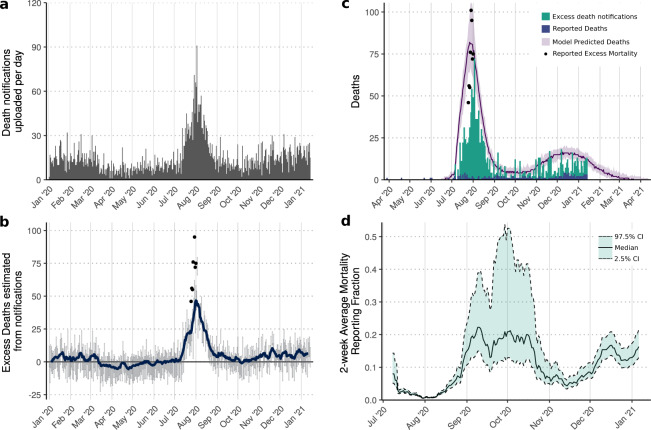
Characterising the dynamics of the second wave in Damascus. In (**a**) we show the number of obituary notifications uploaded daily to the community maintained ‘Damascus Mortality’ Facebook page. In (**b**), the daily inferred excess death notifications are shown, with error bars showing the 95% CI and the blue line indicating the weekly average excess deaths (*n* = 100). In (**c**) model fits (purple, median and 95% CI in shaded region) to the notification excess death data (vertical green bars) are shown under the assumption that 40% of total COVID-19 deaths are ascertained by the notification excess deaths, which correctly captures the excess mortality reported by the Syrian government between 25 July and 1 August (black dots). The model-predicted COVID-19 deaths in (**c**) are significantly greater than the official reported COVID-19 deaths (blue bars), with the reporting fraction (official deaths divided by model-predicted deaths) since July shown in (**d**). In (**d**) the 2-week rolling average reporting fraction is shown, with the 95% CI shown between the dashed lines.

We scraped the number of death notifications uploaded to the Facebook page by 13 January (Fig. 5a). A modest increase in excess deaths estimated from death notifications was observed starting in the second half of November (Fig. 5b). The increase, however, was significantly smaller than observed during the peak in July and August, suggesting that the ascertainment of COVID-19 mortality in Damascus has increased since the first wave. To estimate the changing reporting fraction, we fitted the model to the death notification data. We assumed that the death notifications capture 40% of the total all-cause mortality (as estimated earlier as the best fit to the 8 days of excess deaths) and estimated the proportion of COVID-19 deaths that have been ascertained throughout the second wave. Using this model, we estimate 3920 COVID-19 deaths (95% CI: 2910–4920) have occurred by 13 January 2021, which equates to 5.75% of COVID-19 deaths in Damascus being reported since the start of the pandemic (Fig. 5c). However, COVID-19 mortality ascertainment has changed considerably throughout the epidemic. Earlier, we estimated that 2% of COVID-19 deaths had been reported by 2 September based solely on the death notifications data reported by this date. However, between 3 September and 13 January, we estimate that 12.6% of COVID-19 deaths in Damascus have been reported. In addition, the ascertainment of COVID-19 deaths throughout the pandemic has generally increased (Fig. 5d), however, it decreased considerably during the observed peak in excess mortality during July and August. Lastly, the obituary notification data suggests that if transmission remains the same that the second wave will not be larger than the first wave and is predicted to fall over the next 3 months (Fig. 5c). Through the use of the death notifications, we are able to highlight the stark difference between the epidemic dynamics suggested by the official reported statistics and that inferred from death notifications.

## Discussion

The apparent absence of high levels of mortality in Syria, given the extent of transmission occurring elsewhere in the Middle East region, is surprising considering the transmissibility of a new pathogen that was both anticipated and borne-out in other countries. Based on government reports of all-cause mortality in the governorate of Damascus over the period 25 July–1 August, we conclude that the reported COVID-19 deaths only capture a small proportion of the true number of deaths due to COVID-19 in the city. Despite the estimate of excess mortality only being available for just over a week, we estimate that 1.25% (sensitivity range 1–3%) of deaths from COVID-19 have been reported in Damascus during this period. As a consequence, it is likely that the epidemic in Damascus is in a much more advanced stage than the reported data would suggest, with ~40% of the population infected during the first wave.

In this analysis we relied on two supplementary and unorthodox measures of COVID-19 burden to inform the ascertainment of deaths reflected in official death statistics: 8 days of excess mortality data and publicly uploaded obituary notifications. These measures have limitations in terms of completeness and representativeness which make estimating the precise extent of spread within Damascus to date impossible. For example, in Supplementary Fig. 6 we demonstrated how an unmitigated epidemic leading to 80% attack rates could explain the official reported data equally well if we assume that deaths are only reported for people who die in hospitals. However, when viewed together they provide a consistent picture of a much higher level of mortality and a more mature epidemic than official data would suggest. Unfortunately, the absence of consistent and reliable estimates of excess mortality is not unique to Damascus and as such alternative data sources are likely to be needed in many settings to understand how widespread COVID-19 may be. Additionally, although the notification data are suggestive of substantial under-ascertainment of deaths throughout the outbreak, alternative data sources such as this are also likely to be subject to changing biases over time which are hard to predict. For example, excess death notifications decreased during April and May, possibly due to the most restrictive COVID-19 suppression measures being enforced during this time. In addition, <0.5% of death notifications were attributed to Christians, which is likely lower than the percentage of Christians estimated to be living in Damascus despite recent reports of Christians leaving the country^39^. Assuming that the proportion of Christians living in Damascus have comparable age and risk profiles to the population in Damascus we would have expected a higher proportion of death notifications attributed to Christians. Understanding these biases and exploring how the pandemic has spread across different demographics is key to leveraging alternative data sources and highlights the considerable benefits of regular and complete reporting of excess deaths.

On original submission of this analysis, we predicted a downwards trajectory of the epidemic after 2 September if transmission stayed the same, while cautioning the need for the reductions in transmission achieved by behavioural changes to be maintained. This prediction was largely accurate, however, from December transmission increased, with the effective reproduction number increasing above 1 and *R*_t_ (reproduction number in the absence of immunity) estimated to have increased to 1.2. It seems unlikely that any country will see transmission return to initial *R*_0_ values in the near future due to greater levels of COVID-19 awareness globally compared to early 2020^40^. However, higher levels of attack rate than estimated in Damascus have been observed in other parts of the world^41^, demonstrating the need for continued awareness. If human behaviour towards COVID-19 relaxes, it remains possible that transmission could increase further with Damascus, despite the high attack rates we estimate to have occurred thus far. This would likely have significant secondary impacts on overall mortality in Damascus. One such impact would be the continued toll on medical personnel who have reportedly been heavily affected by the epidemic, with reports of at least 60 Syrian doctors, mostly in Damascus, dying from COVID-19 by 18th August 2020^42^. High levels of healthcare worker infection and mortality would further decrease the limited capacity to effectively treat COVID-19 patients in all areas of Syria, with UN reports of up to 70% of health-workers having left Syria or been killed during ongoing conflict^43^.

In our approach we have relied on excess mortality data, which is dependent on having reliable estimates of baseline mortality, which we sourced from previous annual estimates. However, there are undoubtedly indirect effects that COVID-19 places on healthcare systems, which are hard to quantify but expected to increase all-cause mortality^44^. However, even when we doubled our baseline (non COVID-19) mortality assumption, we still estimate considerable under-ascertainment of COVID-19 deaths, with a maximum of 3% of such deaths being reported during the first wave. Another assumption was our use of age-stratified IFRs taken from Verity et al.^34^ which assume a standard of healthcare comparable to that available in China at the beginning of the pandemic. We explored this assumption in our sensitivity analysis and introduced scenarios in which poorer health outcomes occurred, e.g. due to insufficient oxygen provision. This did not change the inferred level of death under-ascertainment, but did yield lower estimated attack rates due to the higher case fatality rates assumed (Supplementary Fig. 9). Given that 8% of functional public hospitals lacked oxygen support^45^ it is highly plausible that the default parameter estimate of attack rates of 39.0% is an upper estimate, and the true value lies closer to the estimate of 30.0% (95% CI: 24.9–34.3%) assuming poorer health outcomes. Further evidence for the lower attack rate could be suggested by the observed second wave, however, either 30% or 40% attack rate is still susceptible to second waves occurring if R_t_ increases. Central to this is an urgent need for testing to be increased, with only 18,238 COVID-19 tests conducted in Damascus as of 24 August 2020 (last date for which total tests conducted in Damascus were reported) (Supplementary Fig. 10). By 19 December, the Syrian Ministry of Health reported ~80,000 COVID-19 tests had been conducted across Syria^46^.

The analysis presented here was only possible because the Damascus governorate office published mortality data at a key time in the Damascus epidemic. Although this was only 8 days of data, the epidemic trajectory after these dates aligns with an initial plateau in reported COVID-19 deaths in Damascus after 2 August. Further support for this epidemic trajectory was suggested by changes in community-uploaded obituary notifications to Facebook from August onwards. Unfortunately, after the first wave in Damascus occurred, a notable rise in reported cases and deaths was observed in other governorates of Syria. This could signal that the large epidemic in Damascus seeded epidemics within other regions. For example, epidemics could have been seeded from Damascus when inter-governorate travel bans were lifted, a pattern observed in other countries^47^. Alternatively, increases in testing capacity that have occurred in Syria could mean that other governorates are at earlier points in their epidemics compared to Damascus for the same number of deaths^21^. Neither hypothesis can be explored effectively without a more reliable mortality data source in other governorate regions, however, reports of overwhelmed health systems in Idlib do suggest that larger epidemics than suggested by official statistics are occurring^38^. While the level of under-ascertainment across Syria may be similar to that in Damascus, this seems unlikely given the heterogeneity in health infrastructure and testing resources. This need for more reliable and regular data streams for measuring mortality, and for transparency in publishing available data, is equally pressing for many conflict-affected settings. However, in the absence of such data, alternative data sources will have to be leveraged to characterise the dynamics of COVID-19 in many parts of the world. Our analysis provides one example of the level of data that must be included if we are to understand the extent to which the COVID-19 pandemic may have occurred unobserved to date.

## Methods

### Model framework and fitting

We extend a previously published^3^age-structured COVID-19 transmission model to fit to daily reported deaths in Damascus governorate (Supplementary Table 4). As of 2 September 2020, 120 COVID-19 deaths had been reported in Syria, of which 60 were in Damascus governorate (Supplementary Fig. 5). During this time, a number of non-pharmaceutical interventions were implemented in Damascus, including curfews, school and work closures and suspension of mosque visits However, most of these are reported to have been implemented prior to 11th May 2020 with many relaxed on 26th May (Supplementary Table 5). As in our previous work modelling healthcare burden in LMICs^48^, we estimate changes in transmission from relative changes in individual-level mobility (and by extension, patterns of mixing and contact relevant to the transmission of respiratory viruses) as quantified in the Google Community Mobility Reports^49^. However, mobility data is only available in 130 countries, which does not include Syria. In countries without mobility data, we estimate mobility using a Boosted Regression Tree model (Supplementary Fig. 11), trained to predict mobility patterns based on government policies, as quantified by the ACAPs Government-measures dataset (Supplementary Table 3)^50^. We then estimate the effect of changes in mobility on SARS-CoV-2 transmission by fitting to the time series of daily COVID-19 deaths, allowing the effect size of mobility to differ after interventions are relaxed to reflect changes in human behaviour in response to COVID-19^51^.

Under-ascertainment is modelled by assuming that only a proportion of the model-predicted deaths on a given day are reported. When fitting the model, we consider the daily time series of deaths as a partially-observed Markov process. We scan across a range of assumed death ascertainments to relate the Markov process to the observed realisations of death. Reported deaths are assumed to follow a negative binomial distribution (with standard deviation equal to 2 to allow for observed over-dispersion of counts) with an expected mean value given by the model-predicted deaths multiplied by the level of under-ascertainment. In this way we make the assumption that the trajectory of reported deaths is informative of the true trajectory of the epidemic after accounting for under-ascertainment.

Model fitting was carried out within a Bayesian framework, using a Metropolis–Hastings Markov Chain Monte Carlo (MCMC) based sampling scheme. We draw 100 parameter sets from the MCMC chain and use these parameters to simulate epidemic trajectories. For each under-ascertainment assumed, we estimate the likelihood of the model fit by calculating the mean log likelihood for the 100 model-predicted deaths during 25 July–1 August compared against the government-reported excess deaths during the same period. Excess deaths are similarly assumed to follow a negative binomial distribution with an expected mean value given by the model-predicted deaths, with standard deviation 2. Full details of the transmission model and model fitting are available in the Supplementary Material.

### Mortality data

Reported daily mortality and incidence data for Damascus was sourced from the Syrian Ministry of Health^52^. Excess all-cause mortality data between 25 July and 1 August was sourced from a statement by the Damascus governorate^28^. Obituary notification data was sourced from a publicly available Facebook page—‘Damascus Mortality’^36^. Duplicated images were removed before identifying the local time and date at which the image was uploaded to the page. We used Google Cloud’s Vision AI^53^ to select death notifications based on being labelled as ‘Text’ AND ‘Document’, with images matching only one identifier manually assessed as a notification. The date of death for each notification was assumed to be the date the image was uploaded after adjusting for the mean delay from recorded death to notification upload, identified from translating a random selection of 250 notifications. The same 250 notifications were used to estimate the proportion of notifications reporting deaths outside of Damascus, which was then subtracted from the total observed daily death notifications. Excess mortality based on notifications was finally calculated by subtracting the baseline number of notifications per day in 2017–2019, estimated for each month to account for seasonality in mortality.

### Data ethics

The Facebook mortality dataset consists of data scraped from a public Facebook page related to the date and timing of photos uploaded to the page. Google’s mobility data consists of aggregated, anonymized sets of data from users who have chosen to turn on the location history setting. Lastly, the all-cause mortality data between 25 July and 1 August 2020 was obtained from the Damascus Governorate’s Official Facebook page, which publically shared details of mortality during this period.

### Reporting summary

Further information on research design is available in the Nature Research Reporting Summary linked to this article.

## Supplementary information

Supplementary Information

Reporting Summary

## Data Availability

All data used in and generated by this analysis are available in an R research compendium at https://github.com/mrc-ide/syria-covid-ascertainment^54^. This includes external datasets: Google mobility data (https://www.google.com/covid19/mobility/) and the ACAPs intervention data (https://www.acaps.org/covid-19-government-measures-dataset).

## Code availability

All software code and analysis scripts are available in an R research compendium at https://github.com/mrc-ide/syria-covid-ascertainment^54^.
