# Supplementary material for: Leveraging community mortality indicators to infer COVID-19 mortality and transmission dynamics in Damascus, Syria
Source: Nat Commun. 2021 Apr 22;12:2394. doi: 10.1038/s41467-021-22474-9 (PMC8062464; doi:10.1038/s41467-021-22474-9)
Supplement: Supplementary file 1 — Supplementary Information [file 41467_2021_22474_MOESM1_ESM.pdf]

Supplementary Information: Leveraging community mortality indicators to infer COVID-19 mortality and transmission dynamics in Damascus, Syria

Oliver J. Watson,*[1] Mervat Alhaffar,[2] Zaki Mehchy,[3] Charles Whittaker,[1] Zack Akil,[4] Nicholas F Brazeau,[1] Gina Cuomo-Dannenburg,[1] Arran Hamlet,[1] Hayley A Thompson,[1] Marc Baguelin,[1,2] Richard G FitzJohn,[1] Edward Knock,[1] John A Lees,[1] Lilith K Whittles,[1] Thomas Mellan,[1] Peter Winskill,[1] Imperial College COVID-19 Response Team, Natasha Howard,[2,5] Hannah Clapham,[5] Francesco Checchi,[2] Neil Ferguson,[1] Azra Ghani[1], Emma Beals,[6,7] Patrick Walker[1]

Affiliations

1. MRC Centre for Global Infectious Disease Analysis, Jameel Institute for Disease and Emergency Analytics, Imperial College London, London, UK
2. Department of Infectious Disease Epidemiology, Faculty of Epidemiology and Population Health, London School of Hygiene and Tropical Medicine, London, UK
3. Syria team, Conflict Research Programme, London Schools of Economics
4. Google Cloud Developer Advocacy, Google, London, UK
5. Saw Swee Hock School of Public Health, National University of Singapore and National University Health System, Singapore
6. European Institute of Peace, Brussels, Belgium
7. Middle East Institute, Washington, D.C.

* Corresponding Author:

Imperial College COVID-19 Response Team:

Samir Bhatt,[1] Bimandra A Djaafara,[1] Christl A Donnelly,[1] Seth Flaxman,[1] Katy A M Gaythorpe,[1] Natsuko Imai,[1] Elita Jauneikaite,[1] Daniel J Laydon,[1] Swapnil Mishra,[1] H Juliette T Unwin,[1] Robert Verity[1]

Supplementary Methods

Supplementary Note 1: Data Sources and Curation

ACAPs Database and Inferred Mobility

We incorporate interventions using mobility data made publically available from Google (https://www.google.com/covid19/mobility/) [1], which provides data on movement in each country and includes the percent change in visits to places of interest (Grocery & Pharmacy, Parks, Transit Stations, Retail & Recreation, Residential, and Workplaces). We assume that mobility changes will reduce contacts outside the household, whereas the increase in residential movement will not change household contacts. Consequently, we assume that the change in transmission over time can be summarised by averaging the mobility trends for all categories except for Residential and Parks (in which we assume significant contact events are negligible). Google mobility data are unavailable for a number of countries, including Syria. For these locations, we use a Boosted Regression Tree model to infer the change in mobility over time. The model is trained using the timing and frequency of government interventions documented in the ACAPs database [2], with additional sources of government interventions not listed in ACAPs sourced from the WHO PHSM database [3], and the World Bank income status of the country. The combined intervention covariate dataset is provided in Supplementary Table 3. The model was fitted using the statistical software R [4] and the dismo package [5], with tree complexity of 8, bag fraction of 0.5, and a learning rate of 0.05. 5-fold cross-validation was implemented to assess overfitting, and error associated with the test and training datasets found to be similar. The inferred mobility is then normalised such that pre-epidemic mobility is equal to 100%.

Mortality and Incidence Data

Reported daily mortality and incidence data for Damascus was sourced from a number of sources. The majority were acquired from the Syrian Ministry of Health daily COVID-19 updates on their facebook page (https://www.facebook.com/MinistryOfHealthSYR). However, a number of historic deaths which are included in cumulative totals were not identifiable from here and were identified from the revision history of the COVID-19 pandemic in Syria Wikipedia page, which has been maintained each day by copying reported deaths and incidence data from the Syrian MoH COVID-19 dashboard [6], which only provides the governorate breakdown of COVID-19 for the current day.

Supplementary Note 2: Transmission Model

To model the dynamics of a SARS-CoV-2 outbreak and its demand on healthcare over time and resultant mortality we use the same SEIR model structure as in Walker et al. [7]. The model is parameterised to match our current best estimates of key parameters determining the natural history and spread of the virus. The model is available as an R package at https://github.com/mrc-ide/squire (Supplementary Figure 1) and is age-stratified, explicitly incorporating patterns of mixing across and between different age groups. The model includes a

treatment cascade that tracks individuals with respect to their disease severity and the indicated treatment option with individuals assumed to access treatment if it is available (Supplementary Figure 2). For all analysis conducted, squire v0.4.34 was used [8], using the deterministic model implementation and 5 initial seeds randomly distributed in the exposed infection class.

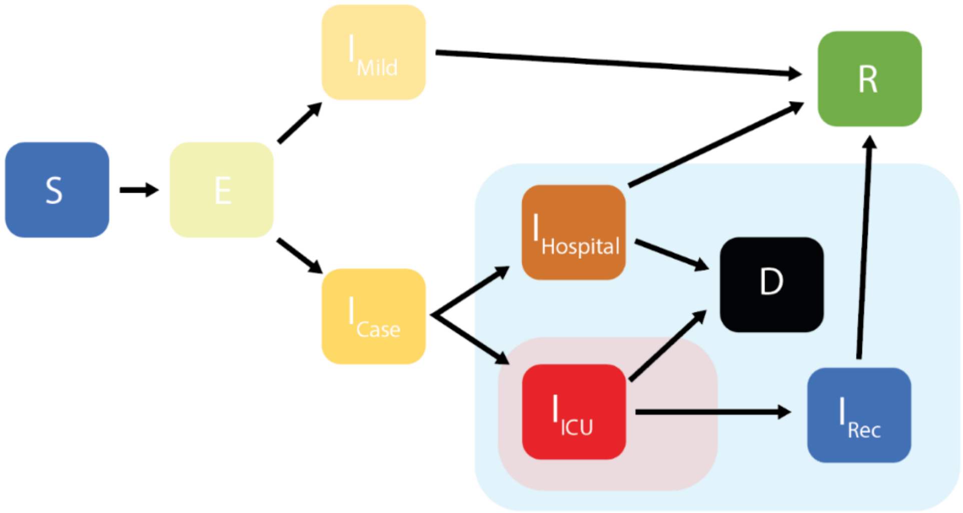

Supplementary Figure 1. Age Structured Compartmental model structure. Individuals in the population begin as susceptible to infection with SARS-CoV-2, **S**. Once infected, determined by an age-specific force of infection estimated from the age-dependent contact rates with infected individuals, and after a latent period of infection, individuals will either develop mild symptoms, $\mathbf{I_{Mild}}$, or severe symptoms requiring hospitalisation, $\mathbf{I_{Case}}$. Individuals in the $\mathbf{I_{Case}}$ compartment are then hospitalised, and will either require high pressure oxygen treatment, which will require a hospital bed, $\mathbf{I_{Hospital}}$, or ventilator support in an ICU bed, $\mathbf{I_{ICU}}$**.** Hospitalised cases either recover, **R**, or die, **D**. Cases in the ICU either die, **D,** or recover, **R**, after spending a period of recovery time in a hospital bed, $\mathbf{I_{Rec}}$. Individuals who suffer mild symptoms, $\mathbf{I_{Mild}}$**,** will recover after a short duration moving into the recovered compartment, **R.** The pale blue box shows compartments related to hospitalisation and occupation of general hospital beds. The pale red box shows compartments related to hospitalisations that occupy an ICU hospital bed.

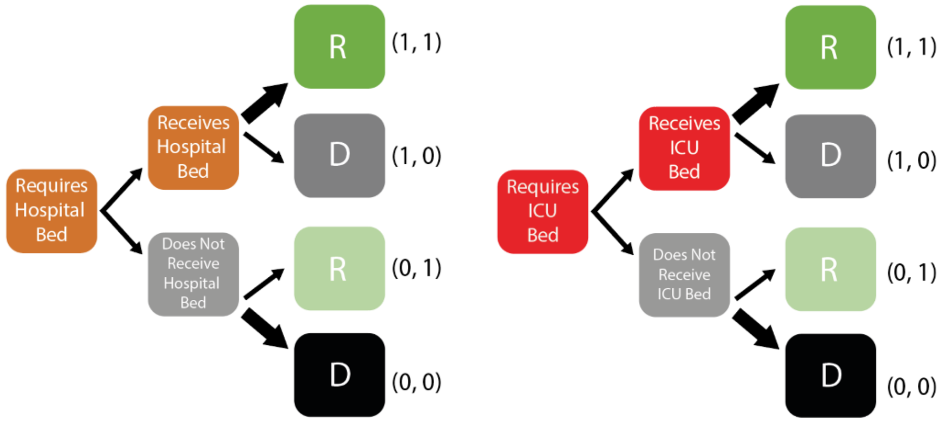

Supplementary Figure 2. Decision tree cascades to capture excess mortality with healthcare capacity being exceeded. Individuals requiring general hospital and ICU beds respectively are assigned available beds (independently of age). Those receiving a bed are subject to a lower probability of mortality than those who do not. Notation to the right hand side of each box describes the compartment in terms of the notation introduced below.

Let S(t,a) denote the susceptible population in age-group a at time t, E_1(t,a) and E_2(t,a) two sequential latent periods of infection, I_{Mild}(t,a) infections that are either asymptomatic or symptomatic but do not require hospitalisation, $I_{Case,0}$ (t,a) and $I_{Case,1}$ (t,a) two sequential states for infections that are symptomatic and will subsequently require hospitalisation. $I_{Hospital,0}$(t,a) and $I_{Hospital,1}$(t,a) are two sequential states for infections requiring a general hospital bed. $I_{ICU,0}$(t,a) and $I_{ICU,1}$(t,a) are two sequential states for infections requiring an ICU bed. $I_{REC,0}$(t,a) and $I_{REC,1}$(t,a) are two sequential states for hospitalised infections in general beds recovering from ICU whilst R(t,a) denotes those that have recovered and are immune to reinfection, and D(t,a) are those that have died from the disease in age-group a. To capture hospital capacity constraints we further split $I_{Hospital,i}$(t,a) and $I_{ICU,i}$(t,a) states (i = 1, 2) states to track those that either receive (1) or do not receive (0) their hospital or ICU bed respectively and through these route either die (0) or recover (1). These additions allow for the different durations of stay in hospital dependent on disease outcome to be captured (Diagram 2). For example, the state tracking those that require a general bed, receive it and go on to die is $I_{Hospital,i}$(t,a,1,0) whilst the state tracking those that require a general bed, do not receive it and go on to die is $I_{Hospital,i}$(t,a,0,0). In the equations below we use the Kronecker Delta function $\delta(.)$ to capture capacity constraints with this equal to 1 if there is capacity (Hospital or ICU) and zero otherwise. Contacts between age-classes are captured using the social contact mixing matrix, c(a, a’), which denotes the rate of contacts between individuals in age-groups a and a’. Age-dependent severity of disease is captured with an age-dependent mortality rate $\mu(a)$. Given that our focus is on short-term dynamics we do not model births, deaths or aging. The age-class of individuals therefore represents their age in 2020. The differential equations describing the model in full are shown below, with the parameter symbols, description and values shown in Supplementary Table 1.

$$\frac{dS(t,a)}{dt} = -\beta \frac{S(t,a)}{N} \sum_{d} c\left(a, a'\right) \left[I_{\text{Mild}}\left(t, a'\right) + I_{\text{Case}}\left(t, a^{\text{r}}\right)\right]$$

$$\frac{dE_2(t,a)}{dt} = \beta \frac{S(t,a)}{N} \sum_{d} c\left(a, a'\right) \left[I_{\text{Mild}}\left(t, a'\right) + I_{\text{Case}}\left(t, a^{\text{r}}\right)\right] - 2\alpha E_1(t,a)$$

$$\frac{dE_2(t,a)}{dt} = 2\alpha E_1(t,a) - 2\alpha E_2(t,a)$$

$$\frac{dI_{Case,0}(t,a)}{dt} = \phi(a)\left(2\alpha E_2(t,a)\right) - 2\gamma_2 I_{Case,0}(t,a)$$

$$\frac{dI_{Case,1}(t,a)}{dt} = 2\gamma_2 I_{Case,0}(t,a) - 2\gamma_2 I_{Case,1}(t,a)$$

$$\frac{dI_{Hospital,0}(t,a,0,0)}{dt} = (1-\delta(H))\mu\left(1-\phi_2(a)\right)2\gamma_2 I_{Case,1}(t,a) - 2\gamma_{3,0} I_{Hospital,0}(t,a,0,0)$$

$$\frac{dI_{Hospital,1}(t,a,0,0)}{dt} = 2\gamma_{3,0} I_{Hospital,0}(t,a,0,0) - 2\gamma_{3,0} I_{Hospital,1}(t,a,0,0)$$

$$\frac{dI_{Hospital,0}(t,a,1,0)}{dt} = (1-\delta(H))\mu\left(1-\phi_2(a)\right)2\gamma_2 I_{Case,1}(t,a) - 2\gamma_{3,0} I_{Hospital,0}(t,a,1,0)$$

$$\frac{dI_{Hospital,1}(t,a,1,0)}{dt} = 2\gamma_{3,0} I_{Hospital,0}(t,a,1,0) - 2\gamma_{3,0} I_{Hospital,1}(t,a,1,0)$$

$$\frac{dI_{Hospital,0}(t,a,0,1)}{dt} = (1-\delta(H))\mu\left(1-\phi_2(a)\right)2\gamma_2 I_{Case,1}(t,a) - 2\gamma_{3,1} I_{Hospital,0}(t,a,0,1)$$

$$\frac{dI_{Hospital,1}(t,a,0,1)}{dt} = 2\gamma_{3,1} I_{Hospital,0}(t,a,0,1) - 2\gamma_{3,1} I_{Hospital,1}(t,a,0,1)$$

$$\frac{dI_{Hospital,0}(t,a,1,1)}{dt} = (1-\delta(H))\mu\left(1-\phi_2(a)\right)2\gamma_2 I_{Case,1}(t,a) - 2\gamma_{3,1} I_{Hospital,0}(t,a,1,1)$$

$$\frac{dI_{Hospital,1}(t,a,1,1)}{dt} = 2\gamma_{3,1} I_{Hospital,0}(t,a,1,1) - 2\gamma_{3,1} I_{Hospital,1}(t,a,1,1)$$

$$\frac{dI_{ICU,0}(t,a,0,0)}{dt} = (1-\delta(H))\mu\left(1-\phi_2(a)\right)2\gamma_2 I_{Case,1}(t,a) - 2\gamma_{4,0} I_{ICU,0}(t,a,0,0)$$

$$\frac{dI_{ICU,1}(t,a,0,0)}{dt} = 2\gamma_{4,0} I_{ICU,0}(t,a,0,0) - 2\gamma_{4,0} I_{ICU,1}(t,a,0,0)$$

$$\frac{dI_{ICU,0}(t,a,1,0)}{dt} = (1-\delta(H))\mu\left(1-\phi_2(a)\right)2\gamma_2 I_{Case,1}(t,a) - 2\gamma_{4,0} I_{ICU,0}(t,a,1,0)$$

$$\frac{dI_{ICU,1}(t,a,1,0)}{dt} = 2\gamma_{4,0} I_{ICU,0}(t,a,1,0) - 2\gamma_{4,0} I_{ICU,1}(t,a,1,0)$$

$$\frac{dI_{ICU,0}(t,a,0,1)}{dt} = (1-\delta(H))\mu\left(1-\phi_2(a)\right)2\gamma_2 I_{Case,1}(t,a)$$

$$\frac{dI_{ICU,1}(t,a,0,1)}{dt} = 2\gamma_{4,1} I_{ICU,0}(t,a,0,1) - 2\gamma_{4,1} I_{ICU,1}(t,a,0,1)$$

$$\frac{dI_{ICU,0}(t,a,1,1)}{dt} = (1-\delta(H))\mu\left(1-\phi_2(a)\right)2\gamma_2 I_{Case,1}(t,a) - 2\gamma_{4,1} I_{ICU,0}(t,a,1,1)$$

$$\frac{dI_{ICU,1}(t,a,1,1)}{dt} = 2\gamma_{4,1} I_{ICU,0}(t,a,1,1) - 2\gamma_{4,1} I_{ICU,1}(t,a,1,1)$$

$$\frac{dIRec,0(t,a)}{dt} = 2\gamma_{4,1} IICU,1(t,a,1,1) - 2\gamma_5 I_{Rec,0}(t,a)$$

$$\frac{dIRec,1(t,a)}{dt} = 2\gamma_5 I_{Rec,0}(t,a) - 2\gamma_5 I_{Rec,1}(t,a)$$

$$\frac{dR(t,a)}{dt} = \gamma_1 I_{Mild}(t,a) + 2\gamma_{3,1} I_{Hospital,1}(t,a,0,1) + 2\gamma_{3,1} I_{Hospital,1}(t,a,1,1) + 2\gamma_{4,1} I_{ICU,0}(t,a,0,1) + 2\gamma_5 I_{Rec,1}(t,a)$$

$$\frac{dD(t,a)}{dt} = 2\gamma_{3,0} I_{Hospital,1}(t,a,0,0) + 2\gamma_{3,0} I_{Hospital,1}(t,a,1,0) + 2\gamma_{4,0} I_{ICU,1}(t,a,0,0) + 2\gamma_{4,0} I_{ICU,1}(t,a,1,0)$$

Supplementary Table 1: Parameter descriptions and values.

Parameter	Symbol	Value	Description
**Epidemiological Parameters**			
Transmission parameter	β	-	Calculated from R_0
Basic reproduction number	R_0	-	Estimated from model fitting
Mean Latent Period	$\frac{1}{\alpha}$	4.6 days	Estimated at 5.1 days [9]. The last 0.5 days are incorporated in the infectious periods to capture pre-symptomatic infectivity
Mean Duration of Mild Infection	$\frac{1}{\gamma_1}$	2.1 days	Incorporates 0.5 days of infectiousness prior to symptoms. In combination with mean duration of severe illness this gives a mean serial interval of 6.75 days [10].
Mean Duration of Severe Infection Prior to Hospitalisation	$\frac{1}{\gamma_2}$	4.5 days	Mean onset-to-admission of 4 days based on unpublished analysis of data from the ICNARC study [11]. Includes 0.5 days of infectiousness prior to symptom onset.
Mean Duration of Hospitalisation for non-critical cases if survive	$\frac{1}{\gamma_{3,1}}$	9.5 days	Based on unpublished analysis of data from the ICNARC study [11].
Mean Duration of Hospitalisation for non-critical cases if die	$\frac{1}{\gamma_{3,0}}$	7.6 days	Based on unpublished analysis of data from the ICNARC study [11].
Mean Duration in ICU if survive	$\frac{1}{\gamma_{4,1}}$	11.3 days	Based on data from the ICNARC study [11] adjusted for censoring.

Mean Duration in ICU if die	$\frac{1}{\gamma_{4,0}}$	10.1 days	Based on data from the ICNARC study [11] adjusted for censoring.
Mean Duration in Recovery after ICU	$\frac{1}{\gamma_5}$	3.4 days	Based on unpublished analysis of data from the ICNARC study [11].
Probability of dying if admitted to critical care	μ	50%	Probability of death from severe infection that is treated based on data from the ICNARC study in the UK [11].

**Age-stratified parameters**				
Age adjusted IFR		0 to 4	0.003%	Age-stratified estimates of the IFR from Verity et al.[1]
		5 to 9	0.002%	
		10 to 14	0.004%	
		15 to 19	0.01%	
		20 to 24	0.02%	
		25 to 29	0.04%	
		30 to 34	0.06%	
		35 to 39	0.09%	
		40 to 44	0.13%	
		45 to 49	0.21%	
		50 to 54	0.44%	
		55 to 59	0.80%	
		60 to 64	1.68%	
		65 to 69	2.65%	
		70 to 74	4.16%	
		75 to 79	6.01%	
		80+	9.42%	

Proportion of infections that require hospitalisation	$\phi_1(a)$	0 to 4 0.001<br>5 to 9 0.001<br>10 to 14 0.001<br>15 to 19 0.002<br>20 to 24 0.005<br>25 to 29 0.010<br>30 to 34 0.016<br>35 to 39 0.023<br>40 to 44 0.029<br>45 to 49 0.039<br>50 to 54 0.058<br>55 to 59 0.072<br>60 to 64 0.102<br>65 to 69 0.117<br>70 to 74 0.146<br>75 to 79 0.177<br>80+ 0.180	Smooth scaling age-stratified estimate of the proportion of infections that require hospitalisation from Verity et al [12].
Proportion of hospitalised cases requiring critical care	$\phi_2(a)$	0 to 4 0.050<br>5 to 9 0.050<br>10 to 14 0.050<br>15 to 19 0.050<br>20 to 24 0.050<br>25 to 29 0.050<br>30 to 34 0.050<br>35 to 39 0.053<br>40 to 44 0.060<br>45 to 49 0.075<br>50 to 54 0.104<br>55 to 59 0.149<br>60 to 64 0.224<br>65 to 69 0.307<br>70 to 74 0.386<br>75 to 79 0.461<br>80+ 0.709	Adjusted estimates from Verity et al [12].

**Adjusted model parameters to capture outcomes related to poorer health outcomes and limited treatment availability**			
Proportion of treated non-critical care cases dying	$\mu(a)$	0 to 4 0.25<br>5 to 9 0.25<br>10 to 14 0.25<br>15 to 19 0.25<br>20 to 24 0.25<br>25 to 29 0.25<br>30 to 34 0.25<br>35 to 39 0.25<br>40 to 44 0.25<br>45 to 49 0.25<br>50 to 54 0.25<br>55 to 59 0.25<br>60 to 64 0.25<br>65 to 69 0.25<br>70 to 74 0.25<br>75 to 79 0.25<br>80+ 0.580	Probability of death from non-severe treated infection. Values here have been adjusted to account for the potentially lower quality healthcare in a LMIC setting "poorer outcomes" in the main text.<br><br>Assumed model defaults from a HIC setting are as follows:<br><br>0.013, 0.013, 0.013, 0.013, 0.013, 0.013, 0.013, 0.013, 0.015, 0.019, 0.027, 0.042, 0.069, 0.105, 0.149, 0.203, 0.580
Mean duration of hospitalisation if require critical care (ICU) but only a general bed is available		1 day	Death is likely to be quicker than in patients in high-income countries.
Probability of dying if require critical care but do not receive it	μ	95%	Probability of death from severe infection that is not treated
Probability of dying if require hospitalisation but no hospital beds and thus oxygen are available	μ	60%	We assume that the outcome is similar to not receiving oxygen.

Supplementary Note 3: Model Fitting

Using the methodological framework developed in the COVID-19 LMIC reports [13], we fit the daily deaths reported by the Syrian Ministry of Health for Damascus [6]. In extension, we now explore a range of under-ascertainment values. Under-ascertainment is modelled by assuming that only a proportion of the model-predicted deaths on a given day are reported. When fitting the model, we consider the time series of deaths, D_t, as a partially-observed Markov process. We scan across a range of assumed death ascertainments to relate the Markov process to the observed realisations of death, which is given by:

$$D_t = NB(\mu.\nu,\ \sigma)$$

where NB is the Negative Binomial distribution, with standard deviation, σ and mean μ multiplied by the level of death under-ascertainment, ν, which is assumed to be constant throughout the epidemic. σ can be expressed as $\sqrt{\mu + \mu^2/r}$, where r is the dispersion parameter and assumed to be equal to 2 to account for overdispersion. The model is fit to D_t by allowing 4 parameters to vary: the start date of the epidemic, t_0, the initial R_0 in the absence of mobility changes, the effect size of mobility on transmission, M_α, and the effect size of mobility on transmission after mobility increases from its minimum, M_ω, which acts on increases in mobility relative to mobility at its minimum, $M(t_m)$. M_ω scales the impact of M_α after the minimum, such that when M_ω is equal to 1, increases in mobility after the minimum will not increase R_t, and when M_ω is equal to 0, there is no decoupling between mobility and transmission, such that R_t will increase with increasing mobility at the same rate as it decreased with decreasing mobility prior to the minimum. In addition, we include a number of pseudo-random walk parameters, ρ_i, which are introduced starting one week after the minimum in mobility, referred to as t_{nd}, which serve to capture changes in transmission that are independent to mobility to reflect changes in human behaviour over time. The equation for the time-varying reproduction number is given by:

$$R_t = R_0 \, . \, f(-M_\alpha . (1 - M(t)) \ - M_\omega . M_\alpha (M(t) \ - \ M(t_m)) \ - \ \rho_1 - \ \rho_2 \dots \rho_n)$$

Where $f(x) = 2\,exp(x)/(1 + exp(x))$, i.e. twice the inverse logit function, which has been used in previous models to capture the impact of mobility data on transmission [14]. $M(t)$ is the inferred mobility throughout the epidemic, in which 1 represents 100% mobility (i.e. no change) and 0 represents 0% mobility. In order to model the changing mobility independent behaviour over time, each ρ parameter is set equal to 0 for each day prior to its start date. For example, ρ_1 is the first mobility independent change in transmission, which starts 7 days after t_{nd} and will be equal to 0 when $t < t_{nd} + 7$. The estimated value for ρ_1 is then maintained for all future time points. ρ_2 is the second mobility independent change in transmission, which starts 21 days after

t_{nd}, i.e. 2 weeks after ρ_1.The last mobility independent change in transmission, ρ_n is maintained for the last 4 weeks prior to the current day to reflect our inability to estimate the effect size of this parameter due to the approximate 21-day delay between infection and death [7].

Model fitting was carried within a Bayesian framework, using a Metropolis-Hastings Markov Chain Monte Carlo (MCMC) based sampling scheme, with adaptive tuning of the proposal implemented during sampling using the Johnstone-Chang optimisation algorithm [15]. All parameter inference results reported here are based on 10,000 iterations, 1,000 of which were discarded as burn-in. The prior distributions and ranges for each parameter estimated used are given in Supplementary Table 2. Centering the prior distribution for the effect size of mobility on transmission at 0 makes no assumption about the effect of mobility on transmission, with a value of 0 leading to R_t being invariant to changes in mobility.

Supplementary Table 2. Prior distributions and ranges

**Parameter**	**Distribution**	**Prior**	**Range**
R_0	Normal	Mean = 3, sd = 1	[1.6, 5.6]
Start Date	Uniform	-	10 - 60 days before first reported death
M_α	Normal	Mean = 0, sd = 3	[-10, 10]
M_ω	Uniform	-	[0, 1]
ρ_i	Normal	Mean = 0, sd = 0.2	[-5, 5]

Supplementary Note 4: Damascus Parameterisation

Our transmission model tracks the number of individuals predicted to develop symptoms sufficiently severe to require hospitalisation, either requiring oxygen support (in less severe cases) or mechanical ventilation (for those with the most severe symptoms). Some 204 intensive care unit (ICU) beds offer mechanical ventilation in Damascus across the private and public health system, with 47% estimated to be available for COVID-19 cases after accounting for expected occupancy due to non-COVID-19 health problems [16]. We assume the number of hospital beds in functional hospitals is equivalent to the number of individuals who can be provided with oxygen support. The Health Resources and Services Availability Monitoring System (HeRAMS) estimated there were 3,245 hospital beds in functional hospitals in Damascus in 2020 [17]. Using estimates of the proportion of beds in public health systems [18], we estimate there are approximately 4,300 beds in functional hospitals in Damascus across both the private and public health systems. Based on inpatient data from previous years [18], we estimate that approximately 1935 (45%) of these beds are available for COVID-19 admissions after accounting for normal hospital demand. Based on UN estimates from the World Urbanization Prospects we assume a population size of 2,394,000 for Damascus governorate [19,20], with an age demographic identical to UN estimates for Syria [19].

Supplementary Note 5: Comparison to excess deaths, sensitivity analysis and model projections

We scan across a range of assumed levels of under-ascertainment of deaths between 0.05% - 20%. For each under-ascertainment value we conduct 1320 individual model fits, each one reflecting a combination of model parameters we vary as part of a sensitivity analysis testing our assumed parameters for Damascus. Full sensitivity analysis values are defined in Supplementary Table 6, but in summary we vary:

- Number of functional hospital beds.
- % of beds occupied by non-COVID-19 patients
- Effective Population Size of Damascus Governorate
- Demographic Profile of Damascus Relative to Syria
- Poorer health outcomes for patients with oxygen indicated due to insufficient oxygen supply.

From each model fit, we sample 100 parameter sets from the MCMC chain, weighted by their log likelihood. Sampled parameters are subsequently used to provide model projections. The likelihood of the model fit against the excess deaths is estimated by taking the mean log likelihood for the 100 model-predicted deaths during 25 July - 1 August compared against the government reported excess deaths during the same period. Excess deaths are similarly assumed to follow a negative binomial distribution with an expected mean value given by the model-predicted deaths, with standard deviation 2.

The best-fitting model using the default parameter set for Damascus (Supplementary Table 4) was subsequently used to provide model projections until the end of 2020. We assume that the mean mobility for the last 7 days is maintained for the remainder of 2020, yielding the final estimated R_t value to be assumed to be constant for the rest of 2020.

Supplementary Note 6: Alternative epidemic trajectories for Damascus

Due to the difficulties in only having 8-days of excess mortality data, there are multiple different epidemic trajectories that could yield the number of excess deaths reported (Supplementary Figure 5). The assumptions made so far to explore the epidemic provide just one trajectory (Supplementary Figure 5a), however, with only 8 days of excess data and a low absolute number of reported deaths it is hard to be certain that this is the correct trajectory. In Supplementary Figure 5 we identify four different scenarios through which the excess mortality could be explained:

A. Current epidemic in Damascus started in February. Due to implemented interventions, transmission remained low until restrictions were relaxed on the 26th May. After this transmission increased resulting in an epidemic. A fixed proportion of all COVID-19 deaths that occur are reported. In this scenario, the excess deaths occur before the peak of the epidemic. This is the default scenario explored in the main analysis.

B. Current epidemic in Damascus started in February. Implemented interventions were less effective than in scenario A and consequently transmission increased earlier in the year. An increasing proportion of all COVID-19 deaths that occur are assumed to be reported over time due to increased testing capacity. In this scenario, the excess deaths occur after the peak of the epidemic.

C. Current epidemic in Damascus started when interventions were relaxed on the 26th May. Due to the absence of interventions, the epidemic proceeded largely unmitigated. A fixed proportion of all COVID-19 deaths that occur are reported. In this scenario, the excess deaths occur before the peak of the epidemic.

D. The same scenario as C), except that only a fixed proportion of COVID-19 deaths that occur within hospitals are reported. Deaths that occur outside of hospitals will always be unreported. In this scenario, the excess deaths occur before the peak of the epidemic.

Each of the four scenarios would reproduce the excess deaths observed between 25 July - 1 August. However, only scenarios A) and D) would also result in both hospital capacity being reached at the end of July (in scenario B hospital capacity would have peaked prior to July) and the ascertained deaths plateauing (in scenario C ascertained deaths would continue to increase during August). For this reason, we repeated our analysis with the assumptions made in scenario D), to demonstrate that with only 8 days of excess mortality, multiple conclusions about the epidemic could be reached. Firstly, we assume that the reported COVID-19 deaths represent only a proportion of all COVID-19 deaths that occur within hospitals. Individuals who die while unable to access a hospital bed are not reported as a death. Secondly, we assume that if more than 20 days occur between reported deaths (the average time from infection to

death [7]), then the epidemic has faded out and new infections are the result of reseeding events in Damascus.

With these alternative assumptions, we again predict significant under-ascertainment, with the best-fitting model suggesting 3% of deaths occurring in hospitals are reported (Supplementary Figure 4). This level of under-ascertainment yielded a good agreement with the three sources of data that we have been evaluating against: reported deaths (Supplementary Figure 6a), excess deaths (Supplementary Figure 6b) and the timing at which hospital capacity is reached (Supplementary Figure 6c). The higher ascertainment predicted in this scenario compared to our original analysis estimate of 1.25% reflects a plateau in reported deaths when health services are beyond capacity. Consequently, the proportion of deaths reported will fall sharply when the health system reaches capacity. Overall, we estimate that 0.28% (95% CI: 0.24% - 0.39%) of total COVID-19 deaths are reported in this scenario. The resultant epidemic trajectory predicts a significantly larger number of excess deaths compared to the original analysis, with 21,255 deaths (95% CI: 15,358 - 24,857) estimated by 2 September 2020 and an attack rate of 83.7% (95% CI: 79.9% - 87.4%) by the end of 2020 (Supplementary Figure 7).

The substantial differences in these trajectories reflect both the difficulties in only having 8 days of excess mortality to characterise the extent of the COVID-19 epidemic in Damascus, and relying on the assumption that a constant relationship exists between the reported deaths and excess mortality. However, there are many reasons why this relationship may be more complex than this, such as changing testing capacity over time (Supplementary Figure 10) and altered treatment-seeking behaviour in response to reports of limited hospital beds [21]. It is for these reasons that we sought an additional source of mortality data in Damascus, turning towards community uploaded obituary notifications.

Supplementary Note 7: Alternative Mortality Data from Death notifications

Due to the multiple trajectories based solely on relating the reported daily deaths to the excess deaths, we sourced an alternative source of mortality data from the “Damascus mortality” Facebook page (https://www.facebook.com/wafiatdimashq) [22]. In Damascus, paper death notifications are affixed to household walls. These death notifications are routinely uploaded by the public to the Damascus mortality Facebook page. The last 10,000 images were available to download from the Mobile uploads and Timeline Photos album pages. Across the two sources, duplicated images were removed before identifying the local time and date at which the image was uploaded to the page. The majority of photos uploaded are scans or photos of death notifications of individuals from Damascus who have recently died, however, a number of images uploaded are either images of the deceased or unrelated images. We used Google Cloud’s Vision AI [23] to select death notifications based on being labelled as “Text” AND “Document”. Where images were identified as either “Text” OR “Document”, we manually inspected images to filter out non notifications, which resulted in 18 039 death notifications (Supplementary Table 7).

From the accompanying html page for each notification, we extracted the date the image was uploaded. The file upload date is likely to have occurred after the date of death. In order to estimate the delay distribution from death to notification upload, a random selection of 100 images were selected and translated to identify the date of death if noted on the notification. The majority of images were uploaded either on the date of death or 1 day after, with all images uploaded within 5 days after death, with a mean delay of 0.49 days. Consequently, we did not adjust for a delay, due to the majority of notifications uploaded on the day of death. Lastly, a small number of notifications are for individuals from Damascus (who have left the country and have relatives in Damascus) and have died outside of the governorate region (inside or outside Syria) but are uploaded to enable those in Damascus who knew the deceased to be informed about the death. Using the same subset of images used to identify the delay distribution from death to file upload, we estimated the proportion of notifications that reflect individuals who died outside of Damascus. From our subset of images, we found that approximately 10% of individuals died outside of Damascus, with the majority dying within Rural Damascus.

In order to use death notifications to model COVID-19, we calculated a baseline notification mortality rate, using deaths reported in 2017, 2018 and 2019. The baseline monthly mortality was estimated as 309 deaths per month, yielding a baseline of approximately 10 notifications/day. We are aware of subtle seasonal dynamics in mortality, with increased mortality during Summer and Winter. In response, we fit a negative binomial distribution to the distribution of daily deaths within each calendar month in 2017 - 2019. Using the inferred distribution, we sampled 100 estimates of the baseline notifications and subtracted this baseline from the count of daily notifications in 2020 to estimate the excess death notifications, which we assume is equal to the number of COVID-19 deaths. The resultant time series was decreased by 10% to account for deaths outside of Damascus, to yield the notification death time series, which we used as an alternative source of data to fit the transmission model to. Lastly, the number of notifications uploaded reflects a fraction of total all-cause mortality in Damascus. 12135, 11680, 12748 deaths were reported in Damascus in 2017, 2018 and 2019 respectively [18]. The adjusted notification time series thus indicates that the notifications capture approximately 27.5% of total all cause mortality between 2017 - 2019. Consequently, the COVID-19 death notification time series used is a conservative estimate of total COVID-19 deaths. When fitting to the excess death notifications we explore a range of assumptions about the assumed fraction of total COVID-19 deaths that are captured by the excess notification deaths. These include the 27.5% estimate based on total historic deaths and 100%, which together provide a lower and upper bound for the analysis. Lastly, we derive a central estimate by fitting to the government reported 8-day excess mortality.

Supplementary Note 8: Data and Code Availability

All software code, data and analysis scripts are available in an R research compendium at https://github.com/mrc-ide/syria-covid-ascertainment.

Supplementary Figures

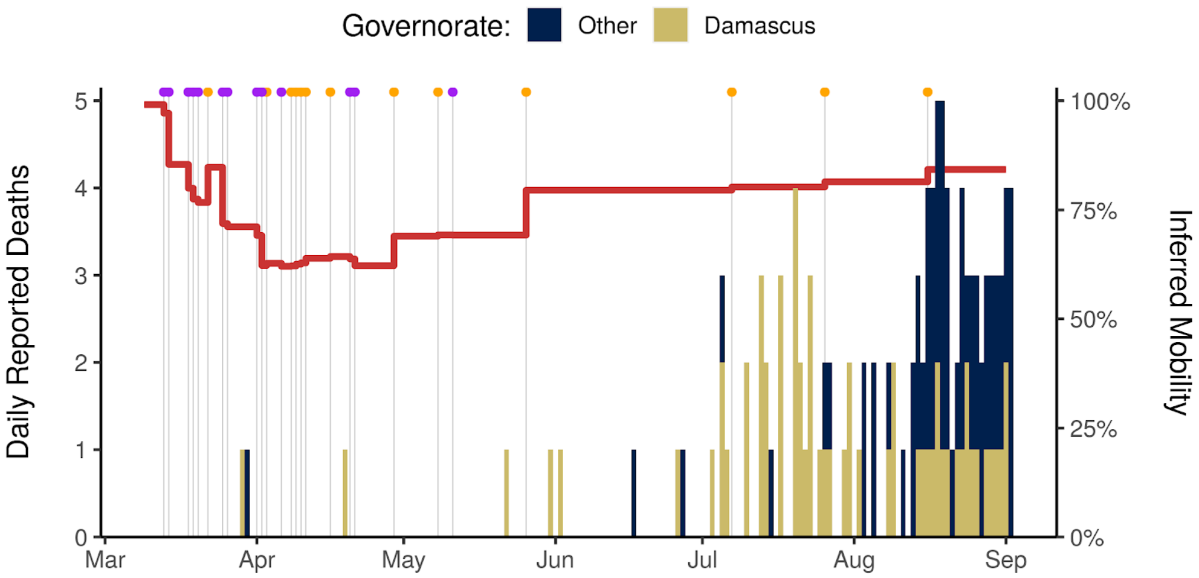

Supplementary Figure 3. Incidence of confirmed deaths due to COVID-19 in Syria up to 2 September 2020. Deaths reported by the Syrian Ministry of Health [6] are shown, with the deaths occurring in the Damascus governorate in gold. The inferred relative mobility pattern for Syria is superimposed with the red line (where 100% indicates the assumed pre-epidemic baseline). The timing of government interventions and policy changes is indicated with vertical lines and points at the top of the y-axis, with purple points indicating when policies were implemented or extended, whereas orange indicate intervention policies being relaxed. As of 2 September, 120 COVID-19 deaths had been reported in Syria, with 60 deaths in Damascus.

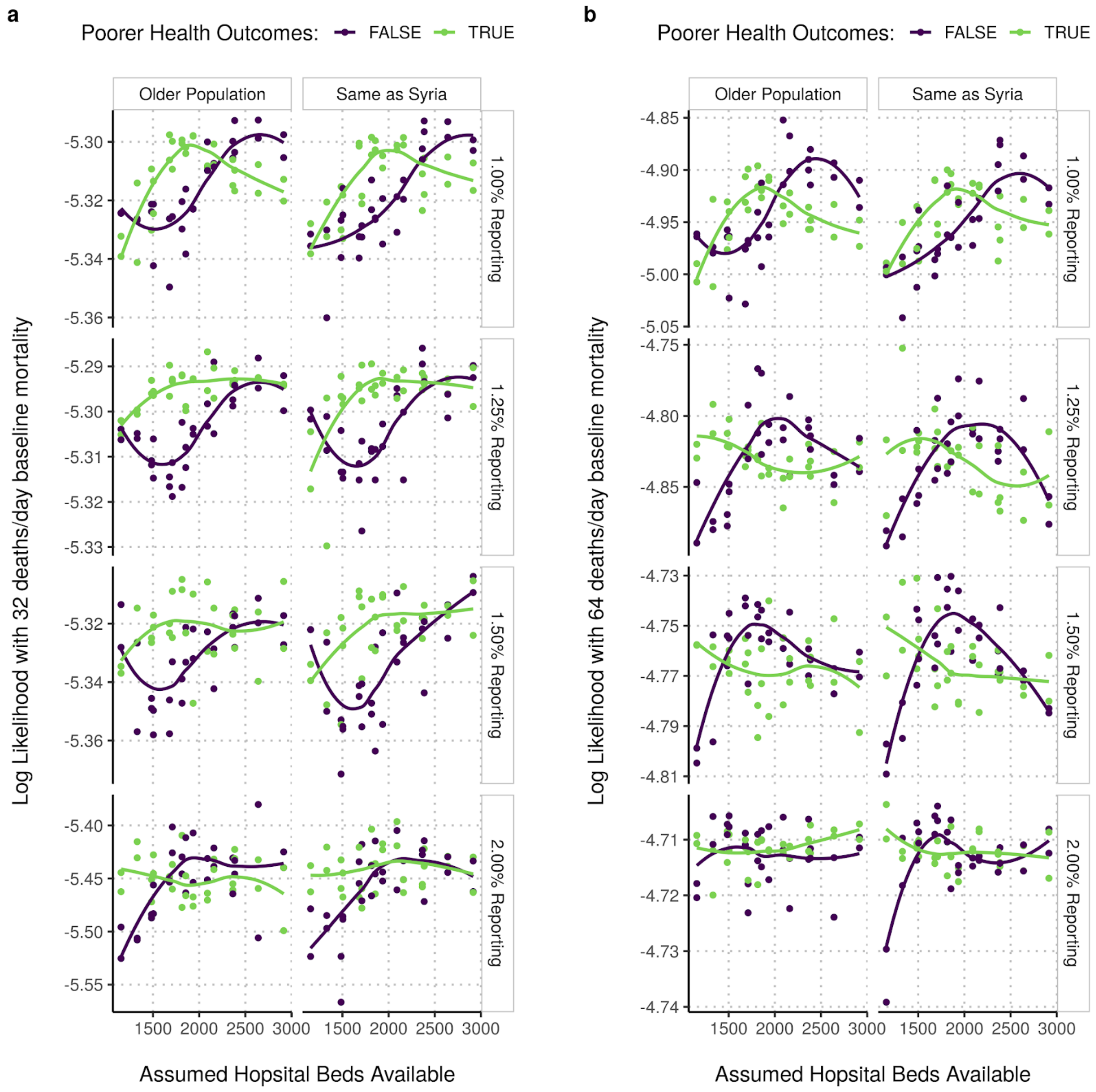

Supplementary Figure 4. Sensitivity analysis of under-ascertainment of deaths. The log likelihood of each model fit is shown, estimated by comparison to excess mortality between 25 July - 1 August 2020, with two baseline mortalities shown in a) and b). The assumed number of beds available after accounting for non-COVID-19 bed demand is shown on the x-axis, with the impact of the assumed health outcomes shown in purple and green. Lastly, the likelihoods shown are subset by the assumed demographic profile used (Older Population compared to Syria or the Same). Model likelihoods are only shown for ascertainment fractions between 1% - 2%, which were identified as being the most likely maximum range for under-ascertainment in Figure 1b for the default parameters. Each point shows a different assumed population size for Damascus governorate.

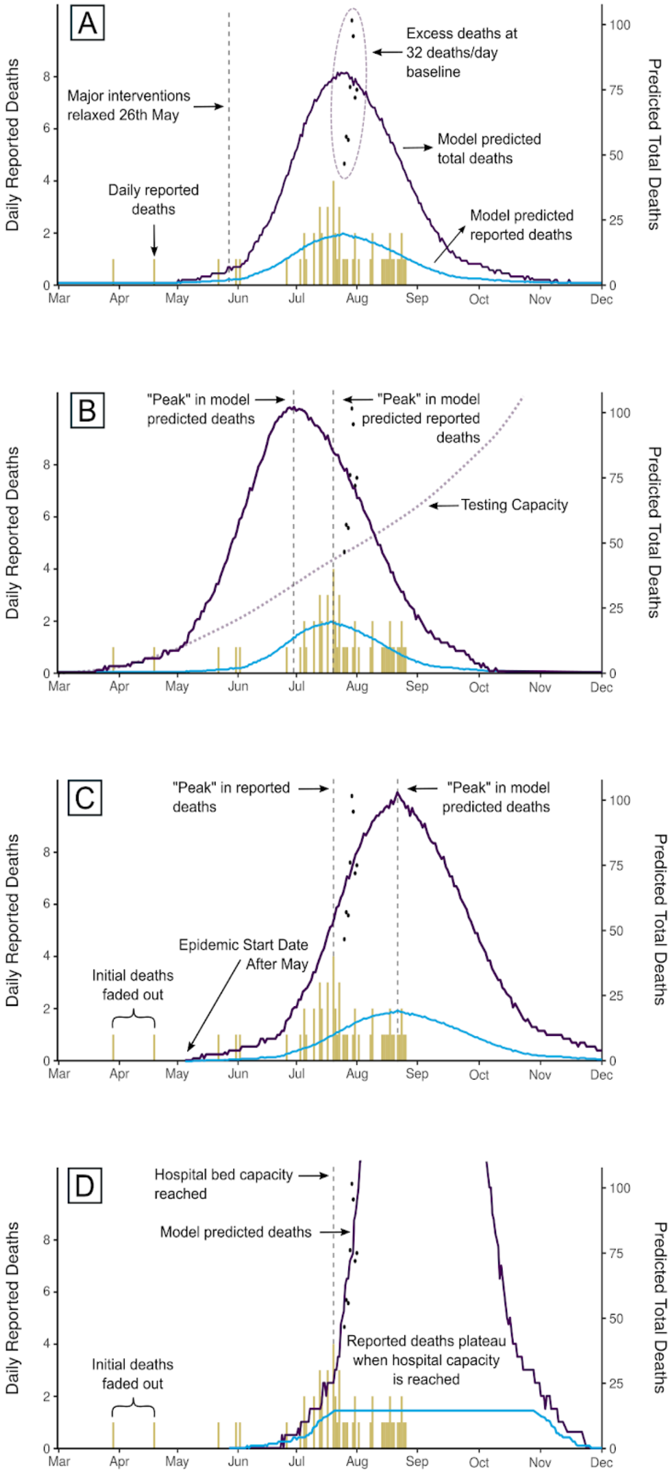

Supplementary Figure 5. Alternative epidemic trajectories. There are multiple epidemics that reproduce reported excess mortality. Our default assumptions yield the trajectory shown in A), with transmission remaining low from March onwards until interventions were relaxed leading to an increase in model-predicted deaths (purple), which given high under-ascertainment results in the model-predicted reported deaths (blue) fitting the reported deaths (gold bars) well. In B), the trajectory passes through the excess deaths after the peak in deaths occurred weeks earlier. However, the scale up in testing capacity, the model-predicted reported deaths still fit the reported deaths well. However, this trajectory would suggest hospital capacity would have been exceeded in June, which does not agree with reported capacity. In C), we assume the initial deaths in March and April stochastically faded out, and the epidemic observed is due to recent importation into Damascus. This results in the trajectory passing through excess deaths before reaching its peak. However, this would result in the peak of model-predicted reported deaths occurring weeks after the observed peak in reported deaths. In D), we assume that deaths can only be reported from individuals who access healthcare and thus will flatten after healthcare capacity is reached. This assumption would both fit the observed reported deaths, while also capturing the timing of healthcare capacity being exceeded and reproducing the excess deaths well.

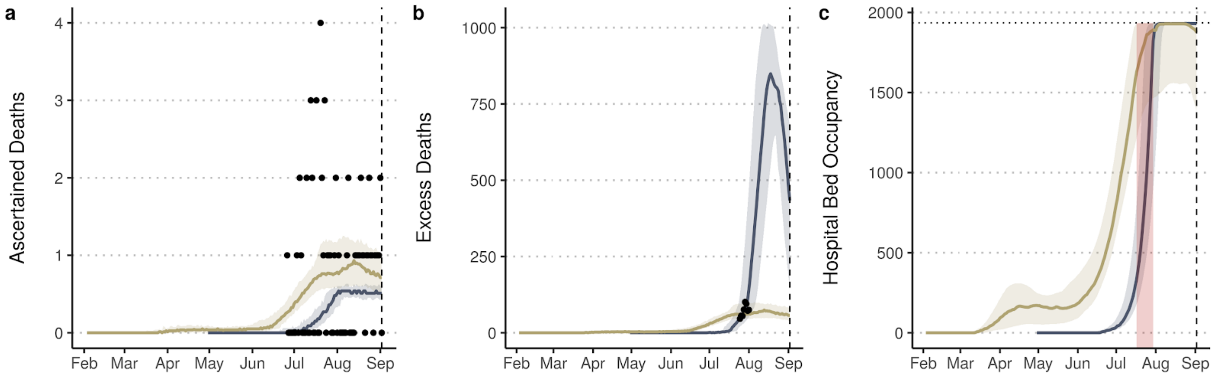

Supplementary Figure 6. Alternative epidemic trajectories for Damascus. The best-fitting model for the default parameters is shown under the assumption that a fixed proportion of all deaths are ascertained (gold) and also under the assumption under that only deaths within the health system may be reported (blue). The model-predicted a) ascertained deaths, b) excess deaths and c) hospital bed occupancy are shown for both epidemic trajectories. In c) the hospital capacity for Damascus is shown with a dashed horizontal line, with the 2-week period in which hospitals were reported to be first at capacity shown shaded in red. In all plots, the median trajectory and 95% confidence interval (shaded region) is shown. A vertical dashed line is shown for 2 September 2020 when the analysis was conducted.

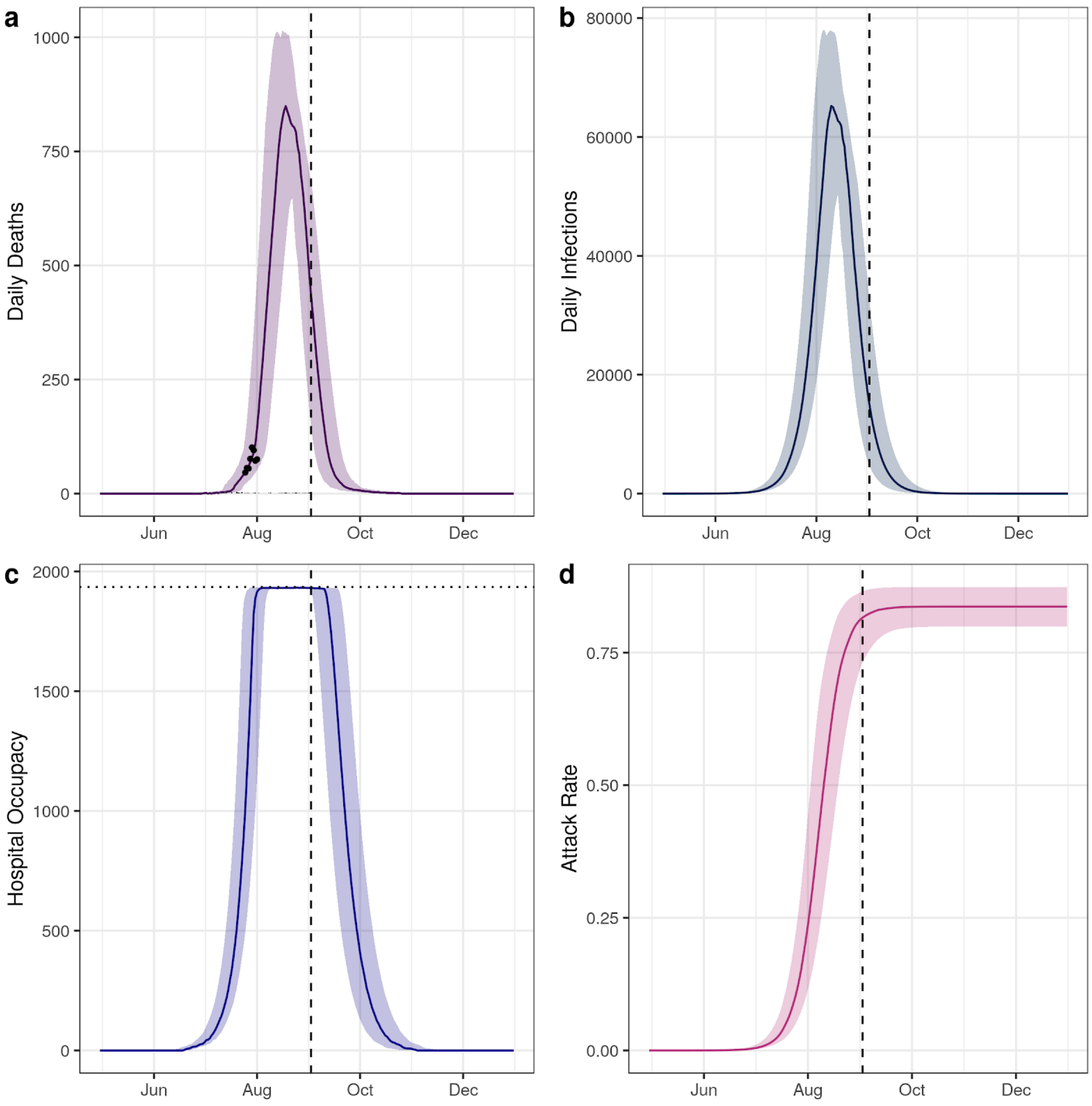

Supplementary Figure 7. Model-predicted deaths, infections, hospital occupancy and attack rates of COVID-19 for Damascus under the assumption that deaths are only ascertained from individuals who access treatment. In a) and b) the reported daily deaths and infections due to COVID-19 respectively are shown, with the estimated excess deaths for a baseline mortality of 32 deaths per day shown in a) as points. In c) hospital occupancy over time is shown, with the dotted horizontal line showing the hospital capacity available. In d) the attack rate in Damascus is shown. In all plots, the median trajectory and 95% confidence interval (shaded region) is shown. A vertical dashed line is shown for 2 September 2020 when the analysis was conducted.

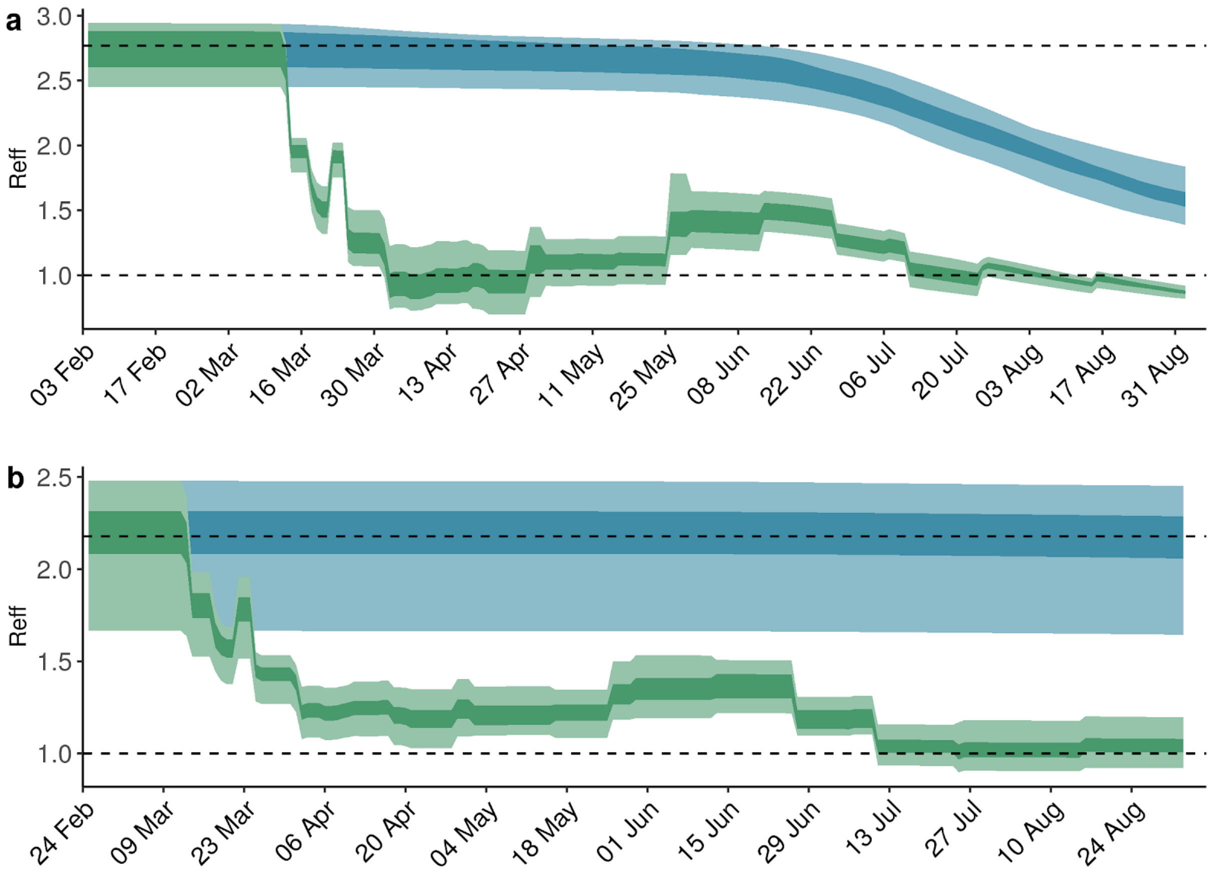

Supplementary Figure 8. Time-varying effective reproduction number, R_{eff}, for Damascus during the first wave. R_{eff} (green) is the average number of secondary infections caused by a single infected person at time equal to t. A horizontal dashed line is shown at R_{eff} = 1. $R_{eff} < 1$ indicates a slowing epidemic in which new infections are not increasing. $R_{eff} > 1$ indicates a growing epidemic in which new infections are increasing over time. Dark green shows the 50% CI and light green shows the 95% CI. The curve in blue shows the predicted decrease in R_{eff} due to increasing immunity in the population resulting from people being infected by COVID-19. Dark blue shows the 50% CI and light blue shows the 95% CI. Individuals infected with COVID-19 are assumed to remain immune within our analysis. In a) the estimated R_{eff} is shown for Damascus with 1.25% under-ascertainment and default parameters and in b) the estimated R_{eff} is shown for Damascus assuming 100% reporting of deaths. The upper horizontal dashed line shows the value of R_{eff} at the beginning of the epidemic, which is equal to the basic reproduction number R_0, highlighting the recent role of immunity in shaping transmission in Damascus in a). Under the assumption of perfect reporting of deaths in b), decreases in transmission will have had to have occurred during a period that occurs just after the majority of interventions were relaxed in Damascus, suggesting under-ascertainment of deaths is a more parsimonious explanation.

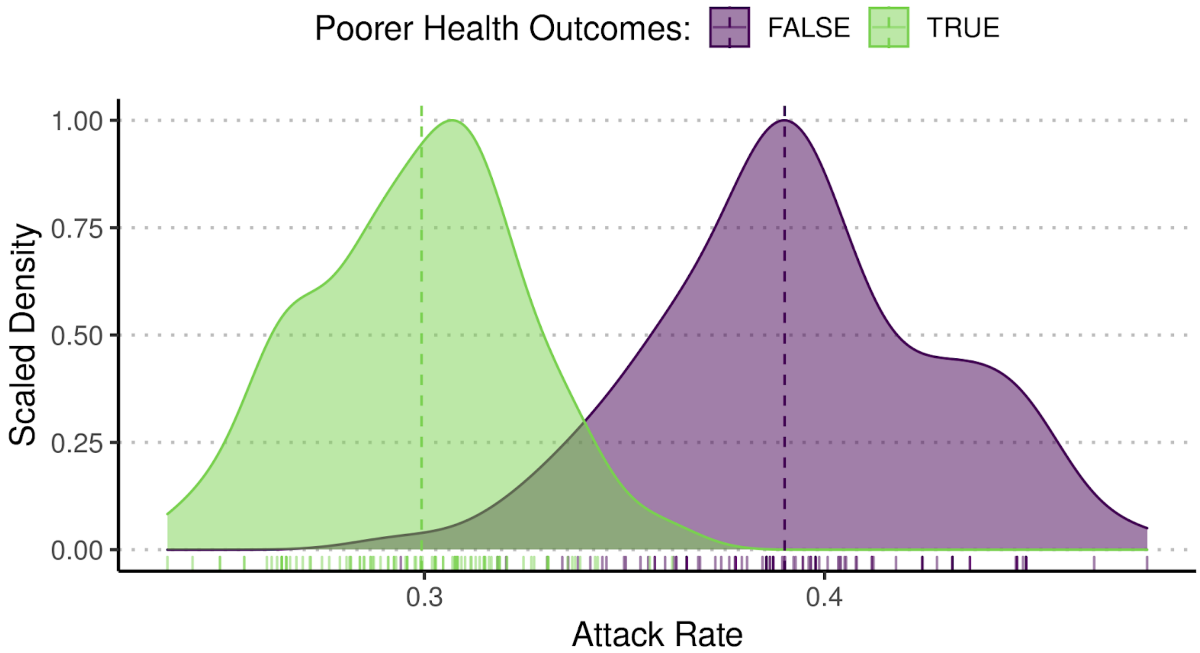

Supplementary Figure 9. Impact of assumed health outcomes on the attack rate by 2 September 2020. The scaled density of sampled attack rates by 2 September 2020 is shown for Damascus with 1.25% deaths reported. When poorer health outcomes compared to estimates in Verity et al. are assumed, a lower overall attack rate is observed with an 0.09 absolute increase in median attack rate.

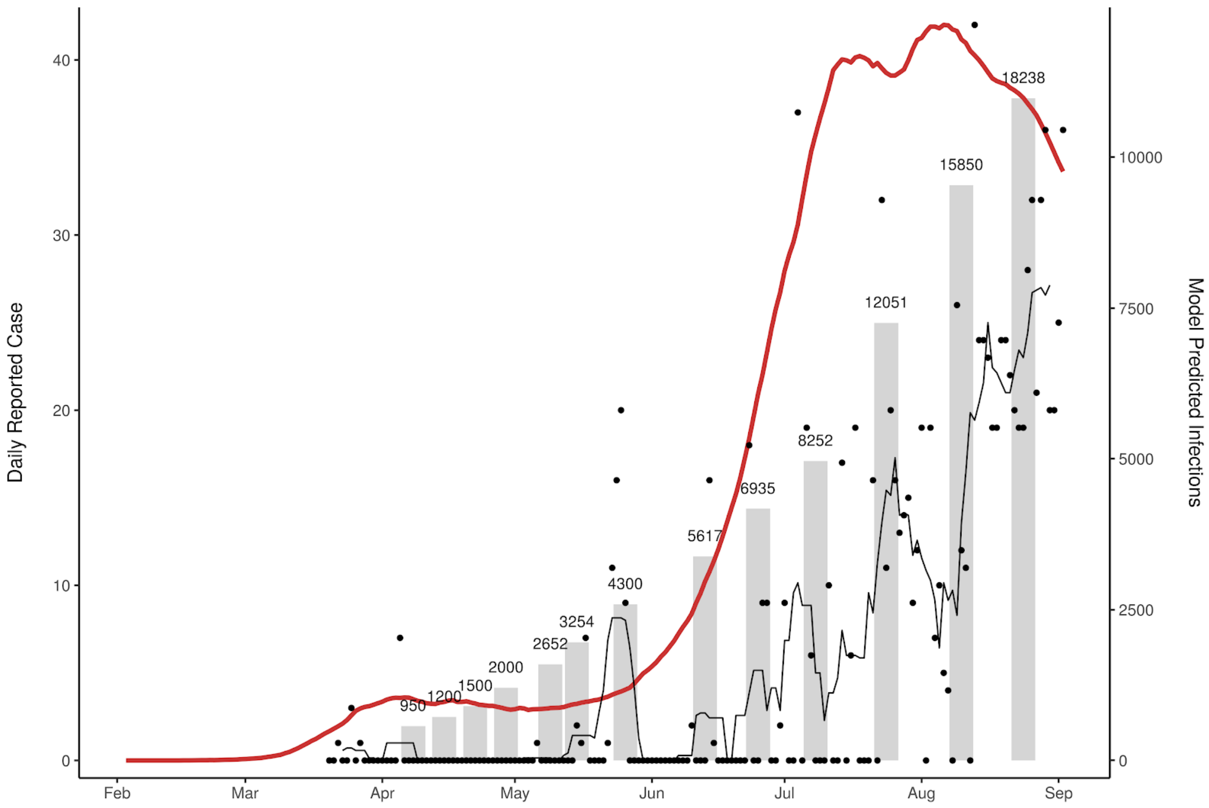

Supplementary Figure 10. Incidence of COVID-19 in Damascus and scale up of testing. Daily reported cases in Damascus are shown with points and the seven-day rolling mean incidence with the black line. The cumulative number of tests reported in Damascus from World Health Organization (WHO) and the Office for the Coordination of Humanitarian Affairs (OCHA) COVID-19 situation updates [24] are shown with vertical grey bars. The model-predicted incidence of infections, including asymptomatic and symptomatic infections, is shown in red.

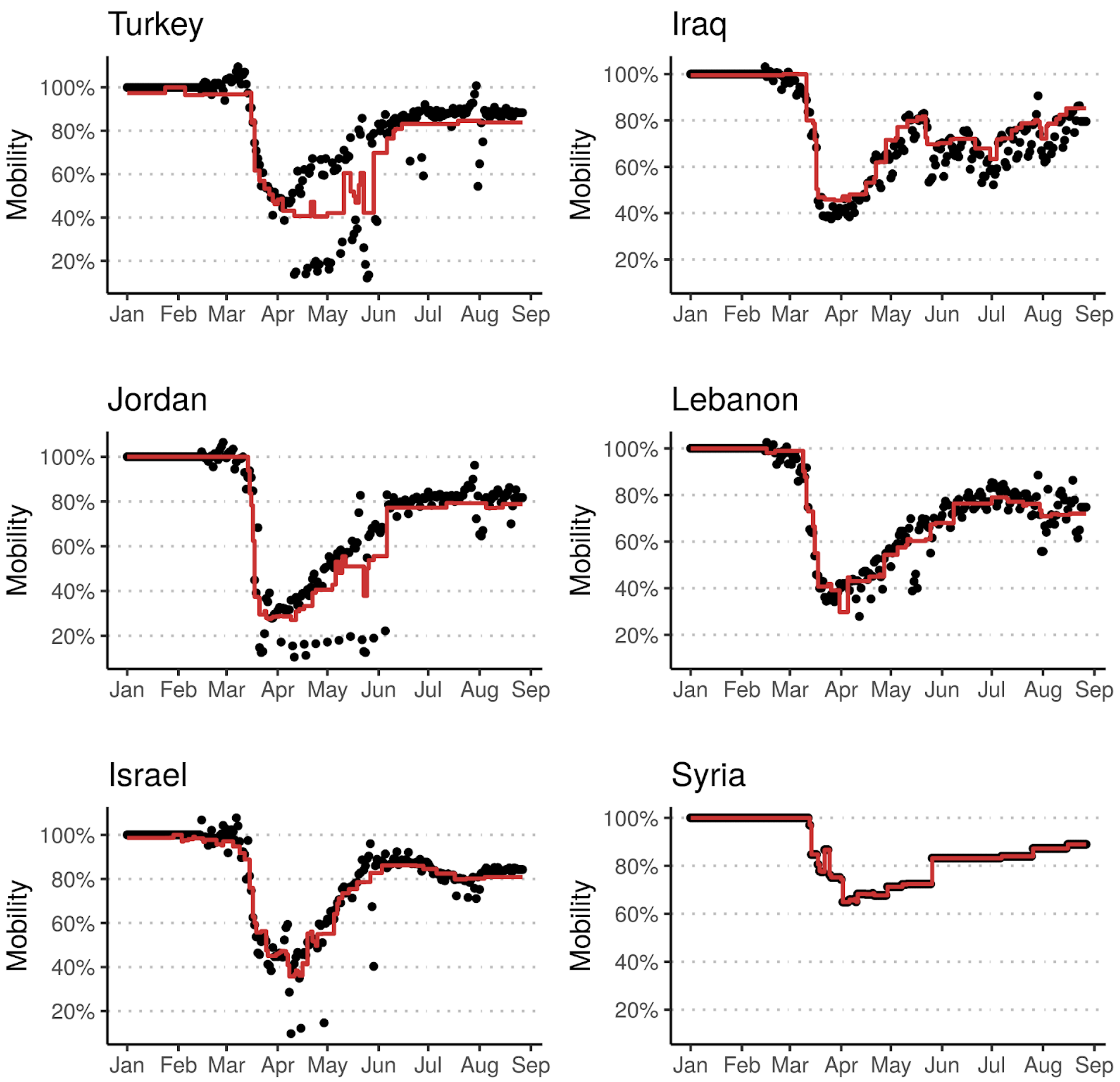

Supplementary Figure 11. Performance of Boosted Regression Tree model for predicting mobility in the Middle East. The points in each plot show the observed national mobility from the Google Mobility Reports. The red line shows the prediction of the Boosted Regression Tree model that infers mobility based on government interventions reported in the ACAPs database. For each country shown, apart from Syria, the true mobility profile is known.

Supplementary Tables

**Supplementary Table 3: Reported all cause mortality in Damascus governorate between 25 July - 1 August 2020** [25].	
**Date**	**Deaths**
2020-07-25	78
2020-07-26	88
2020-07-27	87
2020-07-28	108
2020-07-29	133
2020-07-30	127
2020-07-31	104
2020-08-01	107

Supplementary Table 4: Daily Reported Deaths in Syria by governorate [6]**.** Table is attached as separate document (supp_table_4_revision.csv)

Supplementary Table 5: Government COVID-19 Intervention Policy data. Date and style of government intervention policy is sourced primarily from the acaps government measures dataset [2] with missing policies sourced from the WHO public health and social measures (PHSMs) database [3]. Table is attached as separate document (supp_table_5.csv)

**Supplementary Table 6: Sensitivity Analysis Details.** All combinations of parameters below were explored leading to 1,320 different model fits conducted. The default parameter values are shown in bold, with % of Deaths ascertained not having any value in bold as this was the investigated parameter we were scanning across.	
**Description**	**Values**
% of Deaths Ascertained	20.00%, 10.00%, 6.00%, 3.00%, 2.00%, 1.50%, 1.25%, 1.00%, 0.50%, 0.10%, 0.05%
Number of functional hospital beds	3 300, 3 800, **4 300**, 4 800, 5 800
% of beds occupied by non-COVID-19 patients	45%, **55%,** 65%
Population Size of Damascus Governorate	**2 392 000**, 4 800 000
Demographic Profile of Damascus Relative to Syria	**Same Age**, Older Population
Poorer health outcomes for patients with oxygen indicated due to insufficient oxygen supply.	**False** (IFR same as Verity et al [12]), True (Increased probability of death from individuals with oxygen indicated who receive oxygen as introduced in Walker et al. [7])
Deaths Prior to 26th June led to epidemic fade outs and epidemic observed in July and August due to reseeding events.	**False**, True (Explored in Alternative Epidemic Trajectories Analysis)

Supplementary Table 7: Facebook death notification data. Death notifications were processed using Google's Vision AI API to filter out images that were not death notifications. Details of the image labelling are shown. notifications that were labelled as both "text" and "document" were identified as notifications. Images labelled as only one of "text" and "document" were manually checked and marked to not be included if the image was not a notification. Table is attached as separate document (supp_table_7_revision.csv)

Supplementary References

1. Google. COVID-19 Community Mobility Reports.
2. #COVID19 Government Measures Dataset. https://www.acaps.org/covid19-government-measures-dataset (2020).
3. Tracking Public Health and Social Measures. A Global Dataset. https://www.who.int/emergencies/diseases/novel-coronavirus-2019/phsm (2020).
4. Team, R. C. & Others. R: A language and environment for statistical computing. (2013).
5. Hijmans, R. J., Phillips, S., Leathwick, J. & Elith, J. dismo: Species distribution modeling. *R package version* **1**, 1–1 (2017).
6. Syrian Ministry of Health. Syrian Arab Republic: registered cases of coronavirus. https://app.powerbi.com/view?r=eyJrIjoiNTA0NWMxZmYtMDJiMC00ZWU0LTllNTktZTViZjYwYThjZmUzIiwidCI6ImY2MTBjMGI3LWJkMjQtNGIzOS04MTBiLTNkYzI4MGFmYjU5MCIsImMiOjh9&fbclid=IwAR2sdJgeMcYgezrTShcyrA3HZmqUB2_cx2PIcJguaWJQ3Rb7RRht-3EquyM.
7. Walker, P. G. T. *et al.* The impact of COVID-19 and strategies for mitigation and suppression in low- and middle-income countries. *Science* **369**, 413–422 (2020).
8. Watson, O. J. *et al. mrc-ide/squire: v0.4.34*. (2020). doi:10.5281/zenodo.4024244.
9. Lauer, S. A. *et al.* The Incubation Period of Coronavirus Disease 2019 (COVID-19) From Publicly Reported Confirmed Cases: Estimation and Application. *Ann. Intern. Med.* **172**, 577–582 (2020).
10. Bi, Q. *et al.* Epidemiology and transmission of COVID-19 in 391 cases and 1286 of their close contacts in Shenzhen, China: a retrospective cohort study. *Lancet Infect. Dis.* **20**, 911–919 (2020).
11. *Intensive Care National Audit & Research Centre. ICNARC report on COVID-19 in critical*

care, 2020.

12. Verity, R. *et al.* Estimates of the severity of coronavirus disease 2019: a model-based analysis. *Lancet Infect. Dis.* **20**, 669–677 (2020).
13. Imperial College COVID-19 LMIC Reports. Version 5. MRC Centre for Global Infectious Disease Analysis, Imperial College London. https://mrc-ide.github.io/global-lmic-reports/.
14. Unwin, H. J. T. *et al.* State-level tracking of COVID-19 in the United States. *medRxiv* (2020) doi:10.1101/2020.07.13.20152355.
15. Johnstone, R. H. *et al.* Uncertainty and variability in models of the cardiac action potential: Can we build trustworthy models? *J. Mol. Cell. Cardiol.* **96**, 49–62 (2016).
16. Gharibah, M. & Mehchy, Z. *COVID-19 pandemic: Syria's response and healthcare capacity*. http://eprints.lse.ac.uk/103841/1/CRP_covid_19_in_Syria_policy_memo_published.pdf (2020).
17. Snapshot on WoS Health Resources and Services Availability Monitoring System (HeRAMS) 2020 Q1 : Jan- Mar - Syrian Arab Republic. https://reliefweb.int/report/syrian-arab-republic/snapshot-wos-health-resources-and-services-availability-monitoring.
18. Statistical Abstract. 2019. Central Bureau of Statistics, Damascus-Syria. http://cbssyr.sy/.
19. World Population Prospects - Population Division - United Nations. https://population.un.org/wpp/.
20. United Nations, Department of Economic and Social Affairs, Population Division (2018). World Urbanization Prospects: The 2018 Revision. https://population.un.org/wup/Country-Profiles/.
21. Syria: Weekly Report 17 – 23 July 2020 - Syrian Arab Republic. *reliefweb* https://reliefweb.int/report/syrian-arab-republic/syria-weekly-report-17-23-july-2020 (2020).
22. Damascus mortality page. Facebook. https://www.facebook.com/wafiatdimashq.
23. Vision AI. https://cloud.google.com/vision.

24. World Health Organization (WHO) and the Office for the Coordination of Humanitarian Affairs (OCHA). Syrian Arab Republic: COVID-19 Update No. Search Results. *reliefweb* https://reliefweb.int/search/results?search=Syrian+Arab+Republic%3A+COVID-19+Update+No.
25. Damascus Governorate mortuary estimates 25/07/2020 - 01/08/2020. Government Organization in Damascus, Syria Facebook page. https://www.facebook.com/damascusgovrnorat/posts/191862302286989 (2020).
